# A Roadmap to Cardiac Tissue‐Engineered Construct Preservation: Insights from Cells, Tissues, and Organs

**DOI:** 10.1002/adma.202008517

**Published:** 2021-05-28

**Authors:** Vasco Sampaio‐Pinto, Jasmijn Janssen, Nino Chirico, Margarida Serra, Paula M. Alves, Pieter A. Doevendans, Ilja K. Voets, Joost P. G. Sluijter, Linda W. van Laake, Alain van Mil

**Affiliations:** ^1^ Department of Cardiology Experimental Cardiology Laboratory University Medical Center Utrecht Utrecht University Heidelberglaan 100 Utrecht 3584 CX The Netherlands; ^2^ Regenerative Medicine Center University Medical Center Utrecht Uppsalalaan 8 Utrecht 3584 CT The Netherlands; ^3^ IBET Instituto de Biologia Experimental e Tecnológica Apartado 12 Oeiras 2781‐901 Portugal; ^4^ Instituto de Tecnologia Química e Biológica António Xavier Universidade Nova de Lisboa Av. da República Oeiras 2780‐157 Portugal; ^5^ Netherlands Heart Institute P.O. Box 19258 Utrecht 3501 DG The Netherlands; ^6^ Laboratory of Self‐Organizing Soft Matter Department of Chemical Engineering and Chemistry & Institute of Complex Molecular Systems (ICMS) Eindhoven University of Technology (TUE) Groene Loper 3 Eindhoven 5612 AE The Netherlands

**Keywords:** antifreeze proteins, cardiac tissue engineering, cryopreservation, cryoprotective agents, heart failure, hypothermic and normothermic preservation, vitrification

## Abstract

Worldwide, over 26 million patients suffer from heart failure (HF). One strategy aspiring to prevent or even to reverse HF is based on the transplantation of cardiac tissue‐engineered (cTE) constructs. These patient‐specific constructs aim to closely resemble the native myocardium and, upon implantation on the diseased tissue, support and restore cardiac function, thereby preventing the development of HF. However, cTE constructs off‐the‐shelf availability in the clinical arena critically depends on the development of efficient preservation methodologies. Short‐ and long‐term preservation of cTE constructs would enable transportation and direct availability. Herein, currently available methods, from normothermic‐ to hypothermic‐ to cryopreservation, for the preservation of cardiomyocytes, whole‐heart, and regenerative materials are reviewed. A theoretical foundation and recommendations for future research on developing cTE construct specific preservation methods are provided. Current research suggests that vitrification can be a promising procedure to ensure long‐term cryopreservation of cTE constructs, despite the need of high doses of cytotoxic cryoprotective agents. Instead, short‐term cTE construct preservation can be achieved at normothermic or hypothermic temperatures by administration of protective additives. With further tuning of these promising methods, it is anticipated that cTE construct therapy can be brought one step closer to the patient.

## Graphical abstract

Successful preservation methods for cardiac‐tissue‐engineered constructs will allow transportation and storage for off‐the‐shelf availability, both critical for translating this technology to clinical use, opening new avenues for the treatment/prevention of heart failure. A theoretical foundation for cardiac‐tissue‐engineered construct preservation is presented, with the aim to provide a roadmap to design safe and robust storage protocols.

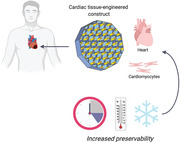

## 1 Introduction

While the number of heart failure (HF) patients is persistently rising, there are still no curative treatments available.^[^ ^1^, ^2^ ^]^ Over 26 million people worldwide suffer from this life‐threatening disease that affects 3–4% of the European population. Therefore, HF has been defined as a global pandemic.^[^ ^3^, ^4^ ^]^ The increase in HF prevalence is the result of population aging and improved survival rates after cardiovascular events such as myocardial infarction (MI).^[^ ^2^, ^5^ ^]^ Following an MI, the heart fails to regenerate adequately, which results in an irreversible loss of cardiomyocytes (CMs) and formation of a noncontractile scar.^[^ ^6^ ^]^ Further adverse remodeling leads to organ dysfunction, which eventually evolves toward advanced HF.^[^ ^4^, ^7^ ^]^ To date, heart transplantation is the gold standard for treating end‐stage HF. However, shortage of availability generates an imbalance between supply and demand of donor hearts.^[^ ^8^ ^]^ Current pharmacotherapy mostly targets symptoms and slows disease progression.^[^ ^2^ ^]^ Left ventricular assist devices (LVADs) can relieve the heart's burden by taking over part of its function. Nevertheless, LVAD placement is no permanent solution due to the associated complications and mortality risk as a result of infections, bleeding, stroke, and right ventricular failure.^[^ ^9^, ^10^, ^11^ ^]^ Hence, available therapeutic approaches are far from curative and do not compensate for the up to 1 billion CMs lost during a MI, nor for the massive cardiomyocyte dysfunction in genetic and acquired cardiomyopathies.^[^ ^12^ ^]^ The deficit in treatment options has motivated the development of regenerative cellular approaches, aiming to functionally restore the damaged myocardial tissue.^[^ ^13^, ^14^ ^]^

Human induced pluripotent stem cells (hiPSCs) provide a source for patient‐specific CMs (i.e., hiPSC‐CMs) that can—after genetic correction if necessary—be exploited for therapeutic purposes. Autologous transplantation of hiPSC‐CMs in a nonhuman primate model of MI showed that these cells could successfully remuscularize the injured myocardium and improve cardiac contractile function, despite the increased incidence of ventricular tachyarrhythmias, which were likely caused by insufficient integration, organization, and maturity of CMs.^[^ ^15^ ^]^ Moreover, cell retention after intramyocardial delivery is extremely low, presumably resulting from cardiac venous drainage and the consequent flow toward the lungs.^[^ ^16^ ^]^ More recently, developments in cardiac tissue engineering enabled the production of various hiPSC‐CM‐based cardiac tissue engineered (cTE) constructs.^[^ ^17^ ^]^ These state‐of‐the‐art constructs consist of hiPSC‐CMs and other cardiac cell types, a collagen‐based hydrogel, and a stretchable microfiber scaffold (both synthetic and natural polymer based), to mimic native myocardial tissue as closely as possible and favor transplanted cells’ retention.^[^ ^18^ ^]^

Despite recent advances, a specific hurdle needs to be overcome to allow clinical application of cTE constructs. Mass production and fast clinical availability are not yet achieved, as no method guarantees reliable construct preservation, hindering both transport and long‐term storage for off‐the‐shelf clinical availability. Therefore, the aim of this review is to provide an overview of currently available preservation strategies and set the stage for the generation of cTE construct‐compatible techniques. Furthermore, development of such techniques is anticipated to simultaneously improve iPSC‐CM and whole‐heart storage, favoring the clinical translation of these cell‐based therapies and increasing the pool of transplantable organs, both contributing to a reduction in HF incidence.

## 2 Ex Vivo Preservation of Biological Samples

Several strategies aiming at prolonging cardiac tissue preservation are currently under investigation. To date, standard whole‐heart preservation (i.e., static cold storage) only grants 4 h of safe heart storage.^[^ ^19^ ^]^ Consequently, a similar time‐window can be expected for clinically relevant large and thick cTE constructs. Two ongoing clinical trials are exploring the potential of iPSC‐CM cell sheets and cTE constructs to remuscularize the myocardium and treat HF.^[^ ^20^, ^21^ ^]^ This highlights the urgent need for the development of efficient construct preservation methods, especially since transportation of cTE constructs requires a minimum of 2‐day storage, and biobanking depends on stable preservation for weeks/months.

**Figure 1:**
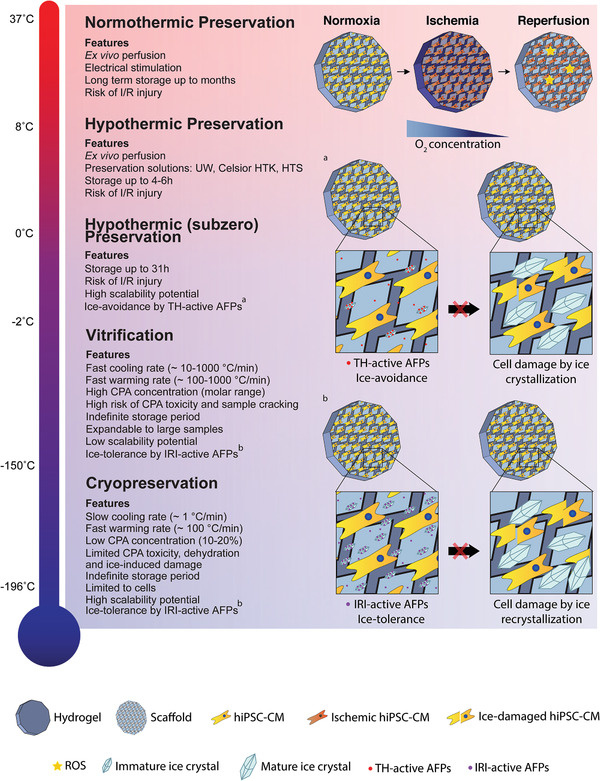
Compilation of the properties, advantages, and drawbacks of each preservation strategy. Preservation techniques can be subdivided depending on their working temperature (normothermic: ≈37 °C; hypothermic: 10 °C to subzero; vitrification: −150 to −160 °C; cryopreservation: −196 °C). At higher temperatures, samples are metabolically active and may experience ischemia/reperfusion (I/R) injury, with reactive oxygen species (ROS) formation, limiting preservation duration. Thermal‐hysteresis (TH) active antifreeze proteins (AFPs) can reduce the freezing point of the preservation solution and allow subzero storage. At lower temperatures, metabolism is halted, allowing for indefinite storage periods, but samples can experience ice‐induced damage. Cryoprotectants (CPAs) are used to prevent ice‐crystal formation and extreme dehydration. Ice‐recrystallization inhibition (IRI) active AFPs prevent ice crystal growth and mechanical damage. Created with BioRender.com.

Preservation strategies can be subdivided depending on the preservation temperature, which are inherently associated with different technical constraints and requirements, as will be explained in the following sections (**Figure** **1**). All methods have the common goal of preventing/delaying the destructive processes that induce ex vivo tissue damage, but may themselves constitute an extra source of harm.

### 2.1 Cryopreservation

Cryopreservation at a temperature of −196 °C is the most widely used cellular preservation technique. At these cryogenic temperatures, sample degradation is virtually stopped allowing in principle for century‐long storage according to Mazur.^[^ ^22^ ^]^ However, intracellular ice crystals may form due to the extremely low cryogenic temperatures, resulting in cell and organelle membrane damage and ultimately cell death. To minimize the likelihood of intracellular ice formation, freezing rates are optimized in cryopreservation protocols to moderate rates of ≈ 1 °C min^−1^. This causes ice to nucleate predominantly in the extracellular space during freezing, since the extracellular space is far larger than the cell volume. As solutes are preferentially excluded from ice crystals, the osmotic pressure rises extracellularly and generates a net flow of intracellular water toward the extracellular space. Consequently, cells dehydrate, which further inhibits intracellular ice formation and generates a rise in the intracellular osmotic pressure. Faster cooling rates (> 10 °C min^−1^) are suboptimal because at these elevated rates water transmembrane movement cannot keep up with the change in osmotic pressure. Thus, it becomes trapped within the cells and results in destructive intracellular freezing.^[^ ^23^ ^]^ Whilst moderate dehydration is fundamental to prevent intracellular ice crystallization, extreme dehydration constitutes an additional source of damage during cryopreservation as it may (partially) unfold, denature or deactivate proteins.^[^ ^24^ ^]^ This motivated the introduction of moderate concentrations (10–20%) of cryoprotective agents (CPAs) during cryopreservation. CPAs increase the intracellular solute concentration and therefore limit water efflux during freezing. Furthermore, CPAs are capable of stabilizing cell membranes and prevent ice crystallization by replacing both intracellular and extracellular water.^[^ ^25^, ^26^ ^]^ (Section 2.3 on cryoprotective agents for additional information).

Vitrification is an interesting alternative to cryopreservation that avoids ice formation altogether. Vitrified tissues are generally stored in vapor‐phase nitrogen (−160 °C) or in electrical freezers (−150 °C), hence at higher temperatures in comparison to cryopreserved samples (−196 °C). To allow for vitrification—the solidification of water into an amorphous glassy state instead of an ordered crystalline state—samples need to be cooled very quickly.^[^ ^19^, ^26^ ^]^ Vitrification of cellular suspensions without CPAs is not possible with the current technology, as this requires an estimated critical cooling rate (CCR) of > 10^7^ °C min^−1^.^[^ ^27^ ^]^ CPAs lower the CCR in a dose‐dependent manner and are used at very high concentrations to enable the vitrification at fast cooling rates of 10–1000 °C min^−1^.^[^ ^19^ ^]^ Disadvantageously however, CPAs also show a concentration‐dependent cellular toxicity, which makes vitrification potentially damaging and more complex compared to conventional cryopreservation.^[^ ^25^ ^]^

Not only cooling but also thawing rates of cryopreserved and vitrified cells and tissues must be controlled. Too slow warming results in significant damage due to ice formation, recrystallization, and sample cracking. Critical warming rates (CWR) which circumvent these complications are usually one or two orders of magnitude larger than CCR.^[^ ^19^ ^]^ This is typically achieved by removing the cell‐containing cryovials from liquid nitrogen and quickly immersing them into a water bath set at 37 °C, preventing ice recrystallization.

### 2.2 Hypothermic and Normothermic Preservation

As cryopreservation has the underlying risk of ice formation and ice‐induced damage, other preservation techniques have been considered. During hypothermic storage (subzero to 10 °C), ice crystal formation is entirely avoided since preservation temperatures do not go beyond the freezing point of the preservation solution. Hypothermic preservation can be particularly useful for the transport of biological material from and to the operating theatre, research institutes or biotechnology companies, being therefore highly relevant for the field of cardiac tissue engineering. During hypothermia, cells experience a reduction of oxygen demand, a slowing of metabolic activity, protein synthesis, transport systems and cell cycle progression.^[^ ^28^ ^]^ Hence, hypothermia can be considered as an approach to maintain cells in a low energy consumption state. Unfortunately, this type of storage is intrinsically restricted to a short period of time as cells still encounter harmful processes.^[^ ^29^ ^]^ ATP production is slowed down, for example, which leads to ion pump impairment and ionic imbalance. This imbalance causes cytoskeleton disassembly and cell swelling, which can be even more deleterious than cryopreservation‐associated cell shrinkage by dehydration.^[^ ^23^ ^]^ Consequently, hypothermic preservation solutions (e.g., University of Wisconsin (UW) solution (or ViaSpan), Celsior, Custodiol histidine‐tryptophan‐ketoglutarate (HTK), or HypoThermosol (HTS)) were designed to prevent the depletion of ATP reserves and counteract these effects, maximizing the time tissues can be safely preserved under these conditions.^[^ ^28^, ^30^ ^]^ Among these, UW solution is considered the most efficient for cardiac preservation.^[^ ^31^ ^]^ To reduce cold‐induced injury, these solutions must meet specific requirements. First, the concentration of ions and cell‐impermeable molecules must result in an ionic and osmotic balance. Second, the formation of free radicals should be prevented. Third, oncotic balance (i.e., protein‐induced osmotic pressure) needs to be preserved. Lastly, preserved samples must be supplied with sufficient energy substitutes.^[^ ^32^ ^]^ An additional approach for expanding hypothermic preservation duration is the lowering of the preservation temperature. By doing so, the metabolic activity of preserved samples is reduced even further (i.e., metabolic rate is halved by each 10 °C reduction),^[^ ^33^ ^]^ which maximizes preservability. Yet, this temperature reduction must not exceed the freezing point of the solution, otherwise ice nucleation and ice‐induced damage initiates.

In contrast to the preceding approaches, normothermic preservation is the only strategy envisaging preservation at close‐to‐physiologic conditions. At normothermia, metabolism and energy consumption are high, therefore preserved samples need to be constantly nourished and oxygenated. For excitable tissues, ex vivo electrical stimulations should also be applied.^[^ ^34^ ^]^

Whereas significant progress has been made regarding the preservation of cells, long‐term preservation of whole‐organs is yet to be achieved. It is expected that data obtained from attempts to preserve CMs, cardiac tissues, and whole‐hearts can be combined to design a cTE construct preservation strategy. Such a strategy should guarantee that preserved cTE constructs maintain the characteristics of their freshly produced counterparts and does not interfere with the safety and potential for clinical application.

### 2.3 Cryoprotective Agents

CPAs are often classified based on their ability to penetrate the cell membrane (i.e., cell penetrating or nonpenetrating agents).^[^ ^26^ ^]^ Cell penetrating CPAs, such as glycerol (GC) and dimethyl sulfoxide ((CH_3_)_2_SO; DMSO), are able to cross cell membranes due to their low molecular weight. These CPAs work by increasing the intracellular concentration of solutes, minimizing the osmotic difference between the intracellular and extracellular environment, thus avoiding extreme dehydration. In parallel, by replacing some of the intracellular water and acting as a solvent, penetrating CPAs prevent intracellular ice crystallization.^[^ ^19^, ^35^ ^]^ Other protective functionalities reported for DMSO include scavenging of reactive oxygen species (ROS) and anti‐inflammatory properties.^[^ ^36^ ^]^

Instead, nonpenetrating CPAs, such as naturally occurring sucrose and trehalose, exert their effects in the extracellular environment. They increase extracellular osmotic pressure, thereby causing water to exit cells, which inhibits intracellular ice crystal formation.^[^ ^35^, ^37^ ^]^ Sugars are primarily used during cryopreservation of spermatozoa and oocytes, where they mainly provide membrane stabilization. Moreover, in vitrification settings, the addition of sugars can be helpful, as sugars have the capacity to raise the glass‐transition temperature (*T*_g_) and lower the CCR and CWR.^[^ ^35^ ^]^

One special class of CPAs are antifreeze proteins (AFPs), which naturally prevent the outgrowth of ice crystals in animals and plants that live in subzero temperature conditions.^[^ ^38^ ^]^ AFPs are ice‐binding proteins that create a thermal hysteresis (TH) gap, lowering the temperature below which water freezes, and/or control ice recrystallization inhibition (IRI), preventing ice crystal growth by fusion of smaller ones and Ostwald ripening.^[^ ^39^ ^]^ Although there is a large variety of AFPs, most studies exploring their potential for cryopreservation focused on fish type I and fish type III AFPs.^[^ ^40^ ^]^ Due to their properties, AFPs allowed the subzero storage of whole rat hearts.^[^ ^41^, ^42^, ^43^, ^44^ ^]^ In contrast to conventional CPAs, AFPs also work in a noncolligative manner and therefore can be used at much lower concentrations, which represents a major advantage as most CPAs are highly toxic.^[^ ^25^ ^]^ Indeed, DMSO, the most used and preferred CPA for cardiac preservation, exhibits cell toxicity. When concentrations exceeded 10% (v/v) at 30 °C, irreversible alterations occurred in the myocardium of rats.^[^ ^45^ ^]^ Moreover, an increase of action potential duration was correlated with myocardium shrinkage in guinea pig papillary muscles subjected to 10% (v/v) DMSO for 30 min.^[^ ^46^ ^]^ Importantly, CPA toxicity is linked to CPA diffusion time. Longer incubation periods are associated with improved cryoprotection but also to increased toxicity. Therefore, a careful optimization of incubation time is required to ensure both safety and protection.^[^ ^47^ ^]^

CPAs can be administered separately or in the form of a cocktail containing multiple CPAs. Synergistic effects have been reported for CPAs administered in combination, as this grants similar cryoprotection while using lower concentrations of each individual CPA, that stay below their toxicity threshold.^[^ ^48^ ^]^ DP6, VS55 (and VS83) and M22 are among the most widely used CPA cocktails (**Table** **1**). Interestingly, addition of other compounds increased further the efficiency of these cocktails. For example, adding 0.6 m sucrose to DP6 and VS55 led to the inhibition of ice formation at a CCR of 1, 5, and 10 °C min^−1^.^[^ ^35^ ^]^ Another study, showed that an enrichment of DP6 with 1,3‐cyclohexanediol produced better results in suppressing ice formation in comparison to sucrose addition.^[^ ^49^ ^]^ To ensure uniform protection, all individual components of the cocktail must be evenly distributed throughout the tissue prior to cryopreservation. The loading time required to achieve such an even distribution for VS83 (combination of DMSO, formamide and propylene glycol) was examined by attenuated total reflection Fourier transform infrared spectroscopy (ATR‐FTIR).^[^ ^50^ ^]^ This revealed that 90% of the DMSO had permeated through the decellularized pulmonary valve after 30 min of incubation, while only 50% of propylene glycol had permeated at that moment. Hence, by combining the permeation data of the different cocktail elements, obtained via real‐time measurements of CPA‐resolved tissue diffusion times by ATR‐FTIR, the estimated loading time required for homogenous distribution of all components can be determined, maximizing tissue protection.

**Table 1:** CPA cocktail composition and physical properties

CPA cocktail	Components [concentration]	Physical properties
DP6	Me_2_SO [3 m], PG [3 m]	*T*_g_: −119 °C; CCR: 40 °C min^−1^
EFS	EG [40% (v/v)], Ficoll 70 [30% (w/v)], sucrose [0.5 m]	–
VS1	Me_2_SO [2.62 m], acetamide [2.62 m], PG [1.3 m], PEG [6% (w/v), 8000 MW]	*T*_g_: −108 to −113 °C; CCR: 5 °C min^−1^
VS2	PG [5.5 m], PEG [6% (w/v)]	*T*_g_: −108 to −113 °C
VS3	GC [6.5 m], PEG [6% (w/v)]	*T*_g_: −108 to −113 °C
VS4	Me_2_SO [2.75 m], PG [1.97 m], formamide [2.76 m]	*T*_g_: −125 °C, CCR: 14.3 °C min^−1^
VS55	Me_2_SO [3.1 m], formamide [3.1 m], PG [2.2 m]	*T*_g_: −123 °C; CCR: 2.5 °C min^−1^; CWR: 50 °C min^−1^
VS83	Me_2_SO [363.2 g L^−1^], formamide [209.3 g L^−1^]; PG [252.6 g L^−1^]	*T*_g_: −119 °C
M22	Me_2_SO [2.855 m], Formamide [2.855 m], EG [2.713 m], *N*‐methylformamide [0.508 m], 3‐methoxy,1,2‐propanediol [0.377 m], PVP K12 [2.8% (w/v)], X‐1000 ice blocker (PVA) [1% (w/v)], Z‐1000 ice blocker (PGL) [2% (w/v)]	*T*_g_: −124 °C; CWR: ≤1 °C min^−1^

GC: glycerol; Me_2_SO: dimethyl sulfoxide (DMSO); PEG: polyethylene glycol; PG: propylene glycol; EG: ethylene glycol; PVP: polyvinylpyrrolidone; PVA: polyvinyl alcohol; PGL: polyglycerol; *T*_g_: glass‐transition temperature; CCR: critical cooling rate; CWR: critical warming rate; Adapted with permission^[^ ^26^ ^]^

## 3 Cryopreservation of Cardiac Samples

### 3.1 Pluripotent Stem Cell Derived Cardiomyocytes

The first experiments to describe long‐term preservation of human pluripotent stem cell derived CMs (hPSC‐CMs) were performed with human embryonic stem cell‐derived CMs (hESC‐CMs).^[^ ^51^ ^]^ hESC‐CMs suspended in cryovials with CryoStor CS10 were cooled to −40 °C using a controlled rate freezer and a cooling rate of 1 °C min^−1^. When cells reached −40 °C, cooling was accelerated to 5 °C min^−1^ until a temperature of −80 °C was achieved. Thereafter, hESC‐CMs were transferred and long‐term preserved in liquid nitrogen (−196 °C). Post‐preservation, a conventional thawing procedure was initiated by submerging cryovials in a 37 °C water bath until all ice melted. With this method, a cell recovery rate of 70–77% was achieved and conservation of cell function was confirmed with cells showing the expected cardiac electrophysiological phenotype and predicted action potential responses to all evaluated pharmacological modulators.^[^ ^51^ ^]^

In another study, hESC‐CMs immersed in supplemented medium containing 10% DMSO and 30% fetal bovine serum (FBS) were successfully cryopreserved in liquid nitrogen.^[^ ^52^ ^]^ The procedure included a pro‐survival step consisting of incubating hESC‐CMs with 10 × 10^−6^ m of rho‐associated kinase (ROCK) inhibitor Y‐27632 prior to freezing, a compound know to prevent dissociation‐induced apoptosis in hPSCs.^[^ ^53^ ^]^ Furthermore, it was demonstrated that hESC‐CMs frozen at day 12 of differentiation exhibited improved survival compared to hESC‐CMs frozen at day 16 of differentiation. In fact, hESC‐CMs preserved at prebeating stage, recovered and formed beating clusters after thawing, whereas cells frozen post‐beating, failed to recover contractility. Importantly, cryopreserved hESC‐CMs maintained their cardiogenic signature (α‐actinin^+^), but transmission electron microscopy revealed increased microstructural damage (e.g., disruption of mitochondrial and nuclear membranes), which was particularly evident in cells frozen at day 16 of differentiation and could have prevented beating recovery.^[^ ^52^ ^]^ As such, it is generally agreed that cryopreservation of hPSC‐CMs should be conducted early in the differentiation protocol to maximize post‐thaw survival.^[^ ^54^ ^]^

**Figure 2:**
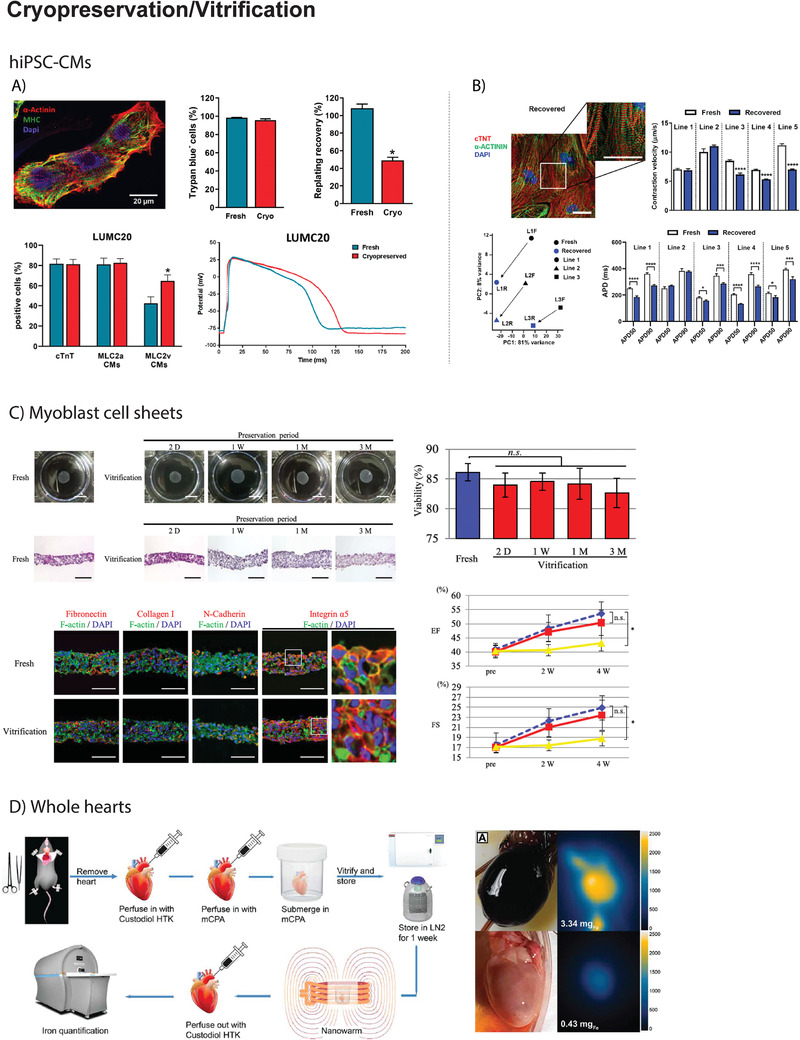
A) In the work by van den Brink et al., hiPSC‐CMs at day 21 of differentiation were cryopreserved and thawed one week after freezing. Viability after dissociation (fresh) or thawing (cryo) was comparable (>95%), but replating efficiency of cryopreserved cells was found to be ≈50% of that of fresh cells. Six days after thawing, hiPSC‐CMs showed a normal cardiac signature (α‐Actinin^+^MHC^+^). In LUMC20 and two other hiPSC‐CM lines, a higher proportion of hiPSC‐CMs expressed the ventricular isoform of myosin light chain (MLC2v), which correlated with an increased duration of the action potentials. Reproduced under the terms of the CC BY license.^[^ ^57^ ^]^ Copyright 2020, The Authors. B) In the work by Zhang et al., hiPSC‐CMs were cryopreserved at differentiation day 23. After thawing, recovered hiPSC‐CMs showed normal cardiac signature (α‐Actinin^+^cTNT^+^). Yet, RNA‐seq analysis revealed transcriptomic alterations in the three analyzed hiPSC‐CM lines and functionally, cryopreserved hiPSC‐CMs showed reduced contraction velocity and shortened action potential duration. Reproduced under the terms of the CC BY‐NC‐ND license.^[^ ^61^ ^]^ Copyright 2020, The Authors. C) In the work by Ohkawara et al., scaffold‐free cell sheets containing human skeletal myoblasts were vitrified. Vitrification did not significantly affect the viability of the cell sheets nor the overall structure, preserving cell–cell adhesions and extracellular matrix composition. In addition, after transplantation onto the heart of a nude rat after surgical induction of MI, vitrified cell‐sheets contributed to an increment of ejection fraction and fractional shortening in the same extent as freshly produced cell sheets. Reproduced under the terms of the CC BY license.^[^ ^62^ ^]^ Copyright 2018, The Authors. D) The publication by Chiu‐Lam et al., reports the first successful vitrification of a rat heart. Briefly, hearts were excised and perfused with Custodiol HTK before being perfused with a magnetic cryopreservation agent (mCPA). After perfusion, the hearts were submerged into the mCPA, vitrified, and stored in liquid nitrogen. After one week of storage, hearts were thawed by nanowarming and subsequently perfused with Custodiol HTK to remove the remaining mCPA. Magnetic particle imaging revealed that mCPA was successfully loaded into the heart before vitrification and removed after thawing. Images on the right illustrate a successful example of rat heart vitrification. Reproduced under the terms of the CC BY‐NC license.^[^ ^72^ ^]^ Copyright 2021, The Authors.

hiPSC‐CMs have become the most important cell source for cardiac disease modeling, cardiotoxicity studies, and cardiac regenerative therapies.^[^ ^55^, ^56^ ^]^ Hence, the direct availability of large numbers of hiPSC‐CMs has become crucial and extensive research regarding their cryopreservation has been conducted. Currently, the two most used cryopreservation solutions for hiPSC‐CM storage are CryoStor CS10 and FBS supplemented with CPAs, most frequently DMSO.^[^ ^54^ ^]^ CryoStor CS10 is a xeno‐free, defined cryopreservation medium that contains 10% DMSO especially developed for freezing extremely sensitive cell types (e.g., hiPSC‐CMs). In contrast, FBS is an undefined solution and batch‐to‐batch variability can be significant. To counteract this issue, it is often replaced by knockout serum replacement (KOSR). Recently, the effects of cryopreservation of hiPSC‐CMs in 10% DMSO and 90% KOSR were evaluated^[^ ^57^ ^]^ (**Figure** **2**A). At 21 days of differentiation into CMs, hiPSC‐CMs were dissociated and cryopreserved via a rate‐controlled temperature decrease (1 °C min^−1^) to −80 °C and then transferred to liquid nitrogen. After one week, hiPSC‐CMs were thawed and incubated at 37 °C. Replating efficiency of cryopreserved cells was found to be approximately half of that of fresh hiPSC‐CMs, indicating that cryopreservation affects cell adhesion/survival.^[^ ^57^ ^]^ Importantly, cryopreserved hiPSC‐CMs showed the same RNA and protein expression levels, electrical activity, and contraction characteristics as nonpreserved hiPSC‐CM. Notably, in three out of four lines tested, a higher proportion of hiPSC‐CMs expressing the ventricular isoform of myosin light chain (MLC2v) was found after cryopreservation, which correlated with an increased action potential duration and expression of ion channels involved in phases 1 and 3 of the action potential. Hence, after correcting for the poor replating capacity of cryopreserved hiPSC‐CMs by seeding approximately twice as many cells per cm^2^, this strategy proved to be adequate for storing hiPSC‐CMs and potentially improve their maturation toward a ventricular subtype.^[^ ^57^ ^]^

To evaluate the impact of cryopreservation media in the recovery and phenotype of cryopreserved hiPSC‐CMs, Miller et al. performed a side‐by‐side comparison of two proprietary (CryoStor CS10 and STEMdiff cardiomyocyte freezing medium) and two common in‐house formulations (90% FBS + 10% DMSO and 90% KOSR + 10% DMSO).^[^ ^58^ ^]^ Cells were cryopreserved at day 14 of differentiation following a slow cooling rate (1 °C min^−1^) and thawed quickly by immersion in a water bath set at 37 °C. Recovery and viability of cryopreserved hiPSC‐CMs did not vary according to preservation solution. Furthermore, at 5‐ and 7‐days post‐thawing, all hiPSC‐CMs showed a similar electrophysiological profile and expression of cardiac Troponin T and MLC2v, respectively.

Overall, the progress in protocols for cryopreservation of hESC‐CMs and hiPSC‐CMs has facilitated cell distribution between different laboratories, research institutions, and countries for comparability experiments aiming at improving consistency in functional assays and addressing the reproducibility issues in biomedical research.^[^ ^59^, ^60^ ^]^ In fact, lack of standardized and comparable studies has limited our understanding of preservation‐induced damage and hampered progress in the field. Hence, additional studies on the evaluation of the effect of cryopreservation and thawing methods in hPSC‐CMs viability and especially functional features are still needed to improve cells’ recovery yields and quality, not only when preserved as single cells but also as 2D monolayers or as 3D cell aggregates. Additionally, state‐of‐the‐art multiomics can depict subtle/early changes in the genetic and proteomic profiles of cells subjected to novel preservation protocols and be used as a tool at the stage of protocol development and optimization. In fact, in a recent study, RNA sequencing revealed an increased expression of cell‐cycle genes in three hiPSC‐CM lines after cryopreservation^[^ ^61^ ^]^ (Figure 2B). Moreover, in comparison to fresh counterparts, cryopreserved hiPSC‐CMs showed a reduced contraction velocity, shortened action potential duration with a propensity to increased beating rate, and were more prone to drug‐induced arrhythmias.

Since the cells’ differentiation stage is a critical variable known to affect the recovery yields after cryopreservation,^[^ ^52^ ^]^ further validation studies using different hPSC lines and different CM differentiation/maturation methods will be needed to confirm the robustness of the (cryo)preservation protocols.

### 3.2 Cardiac Constructs

To date, no research has been conducted focusing on the cryopreservation of cardiac constructs. A possibly comparable example can be found in the vitrification of scaffold‐free cell sheets containing human skeletal myoblasts^[^ ^62^ ^]^ (Figure 2C). These cell sheets were subsequently transplanted onto the heart of nude rats subjected to MI and contributed to an increment of the ejection fraction (EF), preservation of left ventricular dimensions, and reduced fibrosis. During storage, myoblast sheets were preserved by a combination of ethylene glycol (EG), sucrose and carboxy poly‐l‐lysine. After thawing with the assistance of a hot plate set at 37 °C, the preservation solution was removed by immersion in Hanks’ balanced salt solution. In spite of a minor increase in apoptosis, the overall viability of preserved cell sheets was not significantly reduced when compared to the fresh counterparts. Furthermore, preservation had no effect on cell–cell adhesion, extracellular matrix (ECM) composition, and mitochondrial structure. Production of pro‐angiogenic cytokines (e.g., hepatocyte growth factor (HGF)) was found to be higher in vitrified myoblast sheets compared to the fresh, which was interpreted as the consequence of increased hypoxia or ice‐crystal injury in the vitrified group.^[^ ^62^ ^]^ Given the positive results obtained in this study, one can speculate whether this protocol could be applied to vitrify cell sheets containing hiPSC‐CMs.

cTE constructs evolved by embedding hiPSC‐CMs into a hydrogel that is strengthened by a synthetic polymeric scaffold. In fact, most hydrogels commonly used in cTE constructs would themselves fail to resist the mechanical forces of a beating heart, but the addition of an auxiliary polymeric scaffold provides adequate mechanical support. Therefore, during cTE construct cryopreservation, it is extremely important to maintain the integrity of polymeric scaffolds during the liquid‐ice phase transition to avoid detrimental effects on 3D constructs and cell detachment. A recent study on polycaprolactone (PCL) and PCL‐chitosan (CS)‐based electrospun, nonwoven polymeric scaffolds demonstrated that their structure and mechanical properties remain virtually unaltered during long term storage.^[^ ^63^ ^]^ Cryopreservation for 7 days at −150 °C of these PCL and PCL/CS materials did not influence fiber morphology (i.e., fiber diameter and surface topography), while only a slight increase in Young's modulus was observed after thawing. This was tentatively attributed to a difference in the degree of crystallinity of the fibers.^[^ ^63^ ^]^ Similar conclusions were drawn regarding the impact of cryopreservation on the structure and mechanics of scaffolds composed of a polymeric mixture of corn starch and polycaprolactone (SPCL). Scaffold architecture, Young's modulus and surface topography remained unvaried independently of scaffold geometry (i.e., porous or nonporous).^[^ ^64^ ^]^ A direct comparison of cell recovery and attachment on polymeric meshes varying in *T*_g_ and fiber orientation revealed that cell recovery of adherent mouse C2C12 myoblasts could be enhanced using scaffolds composed of fibers of low‐*T*_g_ polymers oriented in a random instead of aligned manner.^[^ ^65^ ^]^ Both characteristics enabled cells to remain (more) attached even upon dehydration during freezing. Scaffolds of polymers with a *T*_g_ below the freezing temperature of the cryopreservation medium (approximately −20 °C for medium supplemented with 10% DMSO) remain more elastic. Therefore, during freezing‐induced cell dehydration, cells do not detach as they shrink, because the polymer matrix can deform as required to maintain cell‐polymer contact. Indeed, cell adhesions were found to persist after cryopreservation for random meshes, whereas cell detachment occurred especially parallel to the long fiber axis of aligned (and tensioned) fiber meshes. Scaffolds composed of low‐*T*_g_ polymers (of sufficiently low molecular weight), such as polyurethane (PU; *T*_g_ −40 °C)^[^ ^65^ ^]^ or PCL (*T*_g_ −60 °C),^[^ ^63^ ^]^ or mixtures of low‐ and high‐*T*_g_ polymers, such as PCL/CS and SPCL meshes, are thus preferred over aligned meshes formed from high‐*T*_g_ polymers such as polystyrene (PS; *T*_g_ 106 °C),^[^ ^65^ ^]^ so that the mesh remains in a rubbery and deformable state while the cells shrink during freezing of the culture medium after which further cooling to storage temperatures below the *T*_g_ induces an increase in stiffness and possibly crystallinity of the scaffold material.

### 3.3 Whole‐Heart

Alike cardiac constructs, efficient cryopreservation of whole‐hearts has not yet been achieved, and most examples of whole‐heart preservation occur by hypothermic preservation (Section 4.2 on hypothermic preservation of whole‐hearts for more information). Notwithstanding, few studies have explored the potential of subzero preservation of whole‐hearts. The rationale behind this strategy consists in achieving a further depression of the metabolic rate while avoiding ice formation, thereby prolonging storage time.

**Figure 3:**
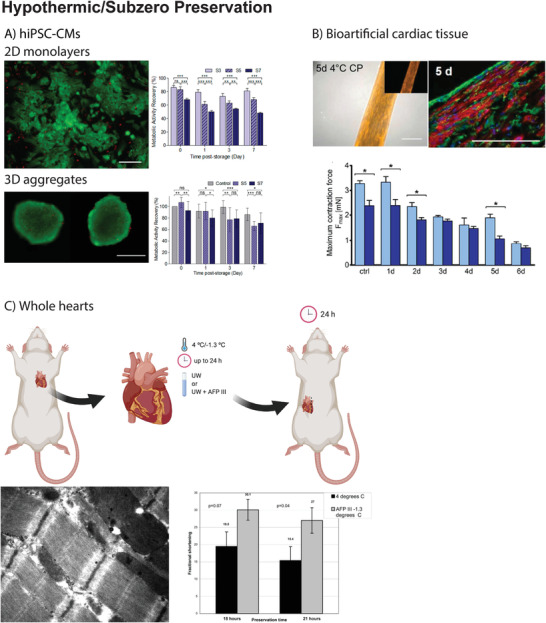
A) In the work by Correia et al., hiPSC‐CMs in 2D monolayers or 3D aggregates were preserved at 4 °C to determine the effect of cell–cell and cell–matrix interactions on preservation success. Cells were preserved in hypothermic conditions for 3, 5, and 7 days (S3, S5, and S7, respectively). After preservation, cells were rewarmed and maintained in culture for up to 7 days. Preservation as 2D monolayers was only feasible up to 3 days, as metabolic activity recovery was significantly compromised for S5 and S7. In contrast, when preserved as 3D aggregates, differences in metabolic activity recovery were less pronounced (e.g., 3D aggregates preserved for 7 days recovered 70% of the metabolic activity 7 days post‐storage). Reproduced with permission.^[^ ^32^ ^]^ Copyright 2016, Wiley. B) In the work by Beckman et al., neonatal rat CMs were mixed with 10% Matrigel and 0.9 mg mL^−1^ rat tail collagen type I in a silicon mold to generate a cardiac construct that was subsequently preserved for 1–7 days at 4 °C. Among the tested preservation solutions, ChillProtec could preserve mitochondrial function after 5 days of preservation (tetramethylrhodamine, methyl ester (TMRM) uptake and staining in active mitochondria, left picture) and cardiomyocyte structure (sarcomeric α‐actinin staining, right picture). In addition, myocardial contraction force was completely preserved after 1 day of preservation at 4 °C but gradually decreased after 5 days of preservation (light blue after 1 day of normothermia, dark blue after 5 days of normothermia). Reproduced with permission.^[^ ^86^ ^]^ Copyright 2018, IOP Publishing Ltd. C) In the work by Amir et al., whole rat hearts were preserved ex vivo at −1.3 °C in UW supplemented AFPIII for a maximum period of 24 h. After preservation, hearts were heterotopically transplanted in the abdomen of a recipient rat. 24 h after the transplant, hearts preserved in the presence of AFPIII showed a superior preservation of cardiomyocyte ultrastructure (membrane, nucleus, and mitochondria) and an improved fractional shortening, when compared to hearts preserved in UW at 4 °C. Reproduced with permission.^[^ ^42^ ^]^ Copyright 2005, Elsevier. Figure 3C top panel was created with BioRender.com.

Reports show that adding fish type I or III AFPs to UW solution allows for the preservation of rat hearts at high subzero temperatures (i.e., −1.3 °C).^[^ ^41^ ^]^ Given the moderate TH activity of these AFPs, hearts could be preserved just below 0 °C without freezing. After a maximum period of 32 h, hearts were rewarmed to 37 °C and connected to a working isolated perfusion system. Supplementation with fish type III AFP improved developed pressures and had beneficial, yet nonsignificant, effects on the survival (i.e., recovery after preservation), heart rate and coronary flow after 24 h at −1.3 °C in comparison to 4 °C in the absence of AFPs.^[^ ^41^ ^]^ Comparison to hearts preserved at −1.3 °C without AFPs with induced ice nucleation was not possible, as these froze and died upon thawing. Most of the hearts (10 out of 12) preserved at 4 °C for 28 and 32 h, failed to beat upon reperfusion and died.^[^ ^41^ ^]^ To test if this type of preservation was compatible with a subsequent transplant, in a follow‐up study from the same group, rat hearts were harvested and preserved for 18 or 21 h in UW at 4 °C or in UW supplemented with fish type III AFP (15 mg mL^−1^) at −1.3 °C, in the presence of nucleation factors, before being heterotopically transplanted into the abdomen of a recipient rat^[^ ^42^ ^]^ (**Figure** **3**C). 24 h after the transplant, hearts subjected to subzero storage with fish type III AFP had superior viability (i.e., assessed by visual inspection of cardiac contraction), improved fractional shortening (FS), fewer apoptotic cells and better conserved myocyte structure.^[^ ^43^ ^]^

Hence, preservation of rat hearts at subzero temperatures was proven possible but limited to 32 h and optimal results are only achieved until 21 h of storage, as myocardial damage started to develop afterward. As such, prolonging cardiac preservation will presumably require lower temperatures, for example those achieved during cryopreservation. However, TH‐active AFPs are only capable of lowering the freezing point of water by a few degrees, which suggests that alternative protective additives are required for long‐term cardiac storage.^[^ ^40^ ^]^

Since cryopreservation is intrinsically linked with the generation of ice‐crystals, preservation media can be supplemented with IRI‐active AFPs to control ice growth and shape.^[^ ^66^ ^]^ Smaller and blunt ice crystals are considered to be less damaging for cells and are more likely to be compatible with cryopreservation of cardiac tissues.^[^ ^40^ ^]^ Regrettably, due to their large size, most AFPs fail to pass the cell membrane, exerting their effect in the extracellular space, having little or no effect in preventing intracellular ice (re)crystallization. As such, covalently binding cell‐penetrating peptides to AFPs could potentially lead to increased cellular uptake.^[^ ^67^ ^]^ This was recently tested using Pep‐1, a cell‐penetrating peptide, to translocate fish type III AFPs intracellularly in A549 cells (adenocarcinomic human alveolar basal cells). Yet, no significant differences were found in the viability of cells frozen in the presence of intracellular or extracellular fish type III AFPs, suggesting that internalization of AFPs may not be (always) required from a viability standpoint.^[^ ^68^ ^]^ Alternatively, to simultaneously achieve extracellular and intracellular cryoprotection, IRI‐active AFPs may be combined with other conventional penetrating CPAs like DMSO, for an efficient cryopreservation of complex cardiac tissues and the whole‐heart. Due to the synergistic effect expected by their combination, lower concentration of both compounds would be required, lowering their toxicity.^[^ ^69^ ^]^ In fact, during muscle preservation, mitochondrial membranes are extremely vulnerable to cold‐induced damage and cryoprotectant toxicity. Mitochondrial function should be preserved during cryopreservation as these are crucial for post‐preservation cell viability and function.^[^ ^70^ ^]^ The capacity of DMSO to protect cardiac mitochondria has been assessed during cryopreservation of rat ventricular muscle fibers.^[^ ^71^ ^]^ Thirty percent DMSO and 10 mg mL^−1^ bovine serum albumin (BSA) were used for cryoprotection prior to cryopreservation. Thawing was performed by transferring samples to a 37 °C water bath. Post‐preservation analysis revealed similar rates of ATP synthesis, ATP/O ratios, and intracellular mitochondrial arrangement, compared to nonpreserved control fibers. Surprisingly, this protocol failed to be replicated by a different group.^[^ ^70^ ^]^ In the latter study, a significant loss in mitochondrial O_2_ consumption was demonstrated in cryopreserved muscle fibers, indicative of mitochondrial damage. Presumably, the addition of metabolic inhibitors could (partly) protect the mitochondria during these extreme conditions.

### 3.4 Vitrification of Cardiac Tissue

Till the present day, vitrification of whole‐hearts was only reported in a single publication by Chiu‐Lam et al.^[^ ^72^ ^]^ (Figure 2D). To achieve it, rat hearts were first perfused with Custodiol HTK and then with magnetic cryopreservation agents (mCPAs) consisting of VS55 supplemented with polyethylene glycol (PEG)‐coated superparamagnetic iron oxide nanoparticles (SPIONs) at a concentration of 5 mg_Fe_ mL^−1^. The perfused heart was then submerged in mCPA and vitrified in a mechanical freezer at a cooling rate of 15 °C min^−1^ before being transferred to liquid nitrogen. After one week of storage, hearts were thawed by nanowarming (Section 3.5. for further information on alternative thawing procedures) using an alternating magnetic field (42.5 kA m^−1^ and 278 kHz, achieving heating rates superior to the CWR of VS55 (50 °C min^−1^)). Finally, SPIONs were removed from the heart by perfusion with Custodiol HTK, which was confirmed using magnetic particle imaging (MPI).^[^ ^72^ ^]^ This pioneering work indicates that vitrification of whole (rat) hearts is possible with the current technology. Yet, recovery after vitrification was merely performed by visual inspection of heart morphology and structure. Further studies are required to evaluate whether vitrified hearts maintain their metabolic and functional properties following rewarming and to validate this approach as a safe alternative for preserving donor hearts for transplantation.

In contrast to heart vitrification, research on kidney vitrification is rapidly progressing and can provide useful information for the development of cardiac‐specific vitrification protocols. In a recent publication, Ehrlich et al. generated a computational model, based on prior experimental work on the vitrification of a rabbit kidney and on the thermal properties of CPAs, to study the thermal conditions required for the vitrification of a human kidney.^[^ ^73^ ^]^ A major issue in kidney vitrification is the high heterogeneity of this organ, which interferes with CPA loading and thermal variation. As such, this model simulates vitrification in a cylindrical container, which allows for a uniform warming when a radiofrequency electrical field is applied. Moreover, the cocktail M22, was selected as cryoprotectant. The modeled protocol consisted of seven consecutive steps: 1) precooling the container and M22 to −50 °C; 2) loading the bare kidney with M22 via vascular perfusion, until the kidney reaches a temperature of −22 °C; 3) transferring the precooled kidney to the precooled container filled with M22; 4) rapidly cooling the container to storage temperatures (−135 °C); 5) storing this system for as long as desired; 6) rewarming the system up to −22 °C by applying a radiofrequency electrical field; and 7) recovering the kidney by diluting the M22 solution via perfusion. This model suggests that the temperature distribution within the kidney is less homogeneous when the system is scaled up from the rabbit to the human size. Importantly, cooling rates remained high enough to prevent ice‐formation even in the 21‐fold larger human kidney. The authors refer some points of improvement such as the adaptation of the cylinder to the shape of the kidney as that would result in improved thermophysical properties.^[^ ^73^ ^]^

Despite several differences between the kidney and the heart, the knowledge generated from computer simulations such as this and empirical observations during kidney vitrification is expected to be useful for the design of protocols for heart and/or cTE construct vitrification.

### 3.5 From Conventional Rewarming to Alternative Thawing Procedures

Most cryopreservation protocols for iPSC‐CMs describe straightforward thawing procedures, where cryovials are transferred to a 37 °C water bath.^[^ ^74^ ^]^ However, this convective warming method is only effective for small samples with volumes ranging from 1 to 3 mL. Thawing is a crucial element of cryopreservation, since ice recrystallization can easily occur, especially in larger samples. Vitrified tissues are no exception and these are particularly prone to recrystallization at temperatures between *T*_g_ and melting (*T*_m_). Therefore, rewarming must be performed as fast as possible, resorting to high warming rates. Importantly, different CPAs have distinct thermophysical properties which define their CWR (e.g., DP6 ≈200 °C min^−1^, VS55 ≈50 °C min^−1^, M22 ≈0.4 °C min^−1^) (Table 1).^[^ ^26^ ^]^ An additional layer of complexity arises from the fact that at high warming rates, the risk of tissue cracking is elevated. Hence, vitrified tissues need to be quickly and homogeneously warmed to simultaneously prevent ice recrystallization and cracking, respectively.^[^ ^19^ ^]^

In this regard, thin metal forms (e.g., copper foam, aluminum foil, or nitinol mesh) were tested in an attempt to provide fast and homogeneous rewarming of vitrified porcine carotid artery loaded with CPAs.^[^ ^47^ ^]^ After exposure to a noninvasive radiofrequency field, the warm metal transferred heat toward the vitrification solution and tissue. After a temperature of −20 °C was reached, the coil was turned off so that the sample could adapt to room temperature. Incubation of carotid arteries with alamarBlue showed that radiofrequency warming was superior to convective rewarming at maintaining tissue viability even when CPA loading was sub‐optimal.

To safely thaw large engineered cardiac tissues, scaling‐up to a volume of approximately 15 mL is required. One technique that allows the efficient rewarming of larger tissues is nanowarming. Manuchehrabadi et al., propose a scalable platform, based on radiofrequency‐excitable mesoporous silica‐coated iron‐oxide nanoparticles (msIONPs) in a VS55 vitrification solution, to achieve warming rates up to 130 °C min^−1^.^[^ ^75^ ^]^ Briefly, msIONPs are added to VS55 before cryopreservation and allow sample thawing by the generation of heat upon radiofrequency excitation. Biocompatibility of msIONPs was ensured by the co‐modification of PEG and trimethoxysilane to the mesoporous coating. The positive effect of msIONPs was demonstrated by thawing porcine arteries and valves after 19–22 h of vitrification in vapor‐phase nitrogen. For small samples (1 mL), nanowarming results were comparable to those achieved by convective warming. However, in larger 50 mL samples, cell viability of convectively warmed tissues declined to ≈ 20% and cracks were visible, which was mainly due to recrystallization caused by an insufficient warming rate. When nanowarming was used, viability and biomechanical properties were preserved.^[^ ^75^ ^]^ Importantly, expansion to 1 L samples, such as whole human organs, might be possible if samples undergo perfusion rather than immersion in VS55 solution containing msIONPs. The disadvantage of these msIONPs is the use of the toxic surfactant cetyltrimethylammonium bromide (CTAB) to generate pores in the particles, which must be removed post‐synthesis and prior their application in cryopreservation. This motivated the development of alternative synthesis routes and/or radiofrequency‐excitable particles, which do not depend on toxic substances. Silica‐coated iron‐oxide nanoparticles (sIONPs) have been introduced, which contain a silica gel instead of a mesoporous silica coating, thereby eliminating the need for CTAB during and its subsequent removal after synthesis. Loading of VS55 with sIONPs resulted in a stable solution, and the post‐thaw removal of sIONPs was superior compared to msIONPs, indicating that sIONPs constitute a more clinical‐compatible alternative.^[^ ^76^ ^]^ Recently, an alternative nanowarming approach, based on the addition of au‐tipped Co_35_Fe_65_ ferromagnetic nanowires (*∅* = 200 nm) to VS55, allowed generation of warming rates of 1000 °C min^−1^, conceding very fast and uniform heating.^[^ ^77^ ^]^

In turn, Lauk‐Dubitskiy et al. proposed an alternative methodology for cryopreservation and rewarming that does not rely on the use of liquid nitrogen and/or CPAs.^[^ ^78^ ^]^ Instead it uses polydimethylsiloxane (PDMS), a cryoprotective heat transfer fluid able to generate a warming rate of 1140 °C min^−1^. The solution was tested during vitrification of heart valves, fragments of the aorta and trachea immersed in precooled low viscosity PDMS‐1 (−75 °C) for 40 s. Thawing was achieved by transferring the tissues to a container with more viscous PDMS‐5 (30 °C) for 1 min. No damage was observed, and cell integrity was maintained. However, cooling and warming rates were again confirmed to be too slow for thick (i.e., >4 mm) samples.

Hence, a critical milestone for studies focusing on alternative and more efficient rewarming techniques is to adapt/optimize current protocols to accommodate larger samples, such as whole human organs. In addition, functional characterization of cryopreserved/rewarmed samples should become a requisite to gain insight into the impact of a preservation protocol on sample physiology rather than survival alone.

### 3.6 Removing Toxic Cryoprotective Agents

Removing CPAs after cryopreservation and before cell or tissue transplantation is essential to prevent toxicity and is typically done by immersion/perfusion with a CPA‐free solution. However, these solutions cause an initial cell swelling as the result of changes in osmotic pressure. Cells regain regular volume when CPAs diffuse toward the administered solution and water accompanies them.^[^ ^79^ ^]^ The European Homograft Bank (EHB) has proposed a dilution protocol for effective DMSO removal from cardiovascular tissues, prior to transplantation, thereby preventing the occurrence of side‐effects in the receiving patients. An independent study tested the efficacy and safety of the method proposed by the EHB in aortic valves, pulmonary valves, descending thoracic aortas and femoral arteries subjected to cryopreservation in 10% DMSO.^[^ ^36^ ^]^ This experiment concluded that DMSO remaining levels ranged from 0.26–1.95 mg kg^−1^ for men and 0.33–2.43 mg kg^−1^ for women, far lower than the 1 g kg^−1^ considered safe in transplanted allografts.

Figure 2 illustrates key publications in the field of cryopreservation and vitrification of cardiac samples.

## 4 Hypothermic and Normothermic Preservation of Cardiac Samples

### 4.1 Pluripotent Stem Cell Derived Cardiomyocytes

Although conventional cryopreservation has been proven useful for the preservation of hiPSC‐CMs, it often fails to prevent extracellular ice formation. Ice is considered to negatively impact the capacity of cells and tissues to establish cell–cell and cell–matrix interactions after thawing.^[^ ^80^ ^]^ Such events can undermine post‐thaw survival and lead to cell loss upon replating. Instead, hypothermic preservation allows the direct preservation of cell clusters or 3D structures. Recently, 2D‐monolayers and 3D‐aggregates of murine iPSC‐CMs and hiPSC‐CMs were preserved to determine the effect of cell–cell and cell–matrix interactions on preservation success^[^ ^32^ ^]^ (Figure 3A). Both cell types were stored for 3, 5, or 7 days at 4 °C in HTS solution. After warming, cells were washed, and kept in RPMI medium + B27 without insulin for a week. During the first 24 h post‐preservation media was supplemented with 10 × 10^−6^ m ROCK inhibitor Y‐27632. Murine iPSC‐CM in 2D‐monolayers or 3D‐aggregates could be stored up to 7 days at 4 °C without significantly compromising their viability or metabolic activity. In contrast, preservation of hiPSC‐CMs as 2D‐monolayers was only feasible up to 3 days, as after this point viability was markedly reduced (i.e., 50% after 7 days of hypothermic preservation). Importantly, cells that did recover from hypothermic storage exhibited a metabolic activity similar to that of the experimental control (i.e., hiPSC‐CMs maintained in culture during the storage period) and this data was also confirmed in hESC‐CMs. Interestingly, when hiPSC‐CMs were preserved in 3D aggregates for 7 days, cell recovery, determined by metabolic activity, reached 70%. No necrotic cells were detected and apoptosis was negligible, and cells maintained their typical phenotype showing normal CM structure/ultrastructure, protein expression profile, and electrophysiological parameters. In addition to providing an efficient hypothermic storage protocol, this study highlights that a 3D architecture seems to confer extra protection against hypothermia‐induced stress, which is likely caused by the establishment of cell‐to‐cell and cell‐ECM interactions.^[^ ^32^ ^]^

These promising results indicate that the structural organization of hiPSC‐CMs in cTE constructs can facilitate their hypothermic preservation.

### 4.2 Whole‐Heart

Currently, hypothermic preservation of hearts for transplantation is limited to 4 h.^[^ ^19^ ^]^ During hypothermic storage, oxygen shortage settles, inducing cold‐induced ischemia. Moreover, during warming, preserved tissues/organs face increased levels of oxygen which leads to the production of ROS.^[^ ^81^ ^]^ As such, prolonged hypothermic preservation provokes ischemia/reperfusion (I/R) injury, with comparable damage to that caused by reperfusion after an MI in vivo.^[^ ^29^ ^]^ Therefore, research on whole‐heart hypothermic preservation aims to prolong storage duration while avoiding preservation‐induced oxygen shortage, oxidative stress, and mitochondrial dysfunction. To date, three studies have studied the effect of protective additives in cardiac hypothermic preservation solutions.

In general, hypothermic conditions lower the metabolic rate and therefore oxygen requirement decreases. Nevertheless, the metabolic rate at 4 °C remains ≈ 10% of that at 37 °C, which explains why hypoxia is still a major cause of cardiac damage during hypothermic preservation. HEMO_2_life, a large nonimmunogenic oxygen carrier comparable to hemoglobin, can carry 156 O_2_ molecules when fully saturated. Earlier, studies proved augmented organ recovery after hypothermic storage of kidneys in UW or HTK solutions enriched with HEMO_2_life.^[^ ^82^ ^]^ Therefore, it was hypothesized that HEMO_2_life could also prolong the time a heart could be safely preserved under hypothermic conditions. Indeed, when HEMO_2_life was added to Celsior, hemodynamic measurements indicated a significantly higher recovery of the left ventricle developed pressure (57 ± 1% vs 45 ± 2%) and coronary flow (7.5 ± 0.7 mL min^−1^ vs 5.4 ± 0.4 mL min^−1^), while no significant differences were found in the recovery of heart rate (88 ± 7% vs 84 ± 4%) when compared to Celsior alone. Ultimately, HEMO_2_life‐mediated additional oxygen supply pushed hypothermic rat heart storage duration up to 8 h.^[^ ^83^ ^]^

In addition, dipeptidyl peptidase 4 (DPP‐4) inhibitors are known for their beneficial effects on the cardiovascular system due to their anti‐inflammatory effects. Experiments with obese and pre‐diabetic rats demonstrated that DPP‐4 inhibitors protected the hearts from I/R injury.^[^ ^84^ ^]^ These findings raised the hypothesis on whether linagliptin, a DPP‐4 inhibitor with enhanced selectivity toward the DPP‐4 enzyme, could serve as a protective additive for cardiac hypothermic storage. To test it, rat hearts were preserved for 9 h at 4 °C in Celsior with (0.25 – 0.75 × 10^−9^ m) or without linagliptin.^[^ ^85^ ^]^ Remarkably, linagliptin could prevent hypothermia‐induced cardiac dysfunction in a concentration‐dependent manner. Hearts treated with linagliptin showed lower left ventricle end‐diastolic pressure, improved coronary flow, and higher left ventricle developed pressure and ± d*p*/d*t*_max_ compared to hearts preserved in Celsior alone. Mechanistically, linagliptin was found to inhibit NOX2, an enzyme involved in ROS production that is upregulated during hypothermic preservation, and subsequently prevent the activation of calmodulin‐dependent protein kinase II and Drp1 translocation to the mitochondria. Mitochondrial Drp1 leads to an opening of the mitochondrial membrane permeability transition pore, mitochondrial fission, and ultimately myocardial dysfunction, all of which could be efficiently repressed by linagliptin supplementation.

Lastly, another molecule expected to diminish hypothermia‐induced I/R injury is propolis, a material collected by bees from buds and barks of trees.^[^ ^81^ ^]^ Propolis is mostly composed of phenolic acids and flavonoids, two bioactive compounds that possess antioxidant properties. The efficacy of propolis was tested by preserving murine hearts at 4 °C for 24 h in Krebs–Hensleit solution with or without propolis (50–250 µg mL^−1^).^[^ ^81^ ^]^ In the propolis‐treated group, post‐storage examination revealed a decrease in oxidative stress (e.g., reduced oxidation of proteins and lipids), a decline in ROS production by mitochondria, and a return of antioxidant enzyme activity to control levels. Additionally, propolis supplementation promoted better preservation of tissue integrity, as shown by a reduced release of lactate dehydrogenase (LDH), creatine phosphokinase and troponin‐I cardiac injury markers to the preservation solution, and histologically by reduced myocardial disarray and cardiomyocyte necrosis. Even though propolis’ contribution for the preservation of cardiac function was not reported, this study indicates the potential of using other natural flavonoids to extend preservation duration.

Figure 3 illustrates key publications in the field of hypothermic preservation and subzero storage of cardiac samples.

### 4.3 Normothermic Preservation

Normothermic preservation is based on maintaining tissues or organs in an environment that resembles as much as possible the endogenous physiological conditions. This type of preservation was applied during the earlier years of organ transplantation and it was possible due to the perfusion with plasma or blood‐based solutions.^[^ ^87^ ^]^ In an attempt to reduce cellular metabolism, perfusion with cold heparinized blood or diluted serum was put forward but frequently led to vascular stasis upon transplantation. In the 1960s, the creation of acellular solutions that mimicked the electrolyte balance of mammalian cells, such as the Collins solutions, permitted efficient preservation of kidneys for up to 30 h at 4 °C in static conditions.^[^ ^88^ ^]^ Static cold storage represented a cost‐effective and convenient alternative that allowed improved tissue matching and sharing of organs between transplant centers and gradually became the gold‐standard for ex vivo preservation of donor organs.^[^ ^89^ ^]^ Several decades later, static cold storage still is the most widely used technique for the preservation of donor organs but the limiting results for whole‐heart preservation (4–6 h) preclude the use of up to 70% of all transplantable hearts.^[^ ^90^ ^]^ Hence, despite technically challenging, normothermic preservation is receiving a renewed attention as a tool to expand the pool of donor hearts. Due to the high metabolic activity at normothermic temperatures, hearts need to be continuously perfused to receive oxygen and essential substrates while eliminating metabolic waste. Yet, stable normothermic preservation would open the possibility to thoroughly assess donor hearts and, if needed, intervene therapeutically with pharmacologic agents and/or gene therapy.

**Figure 4:**
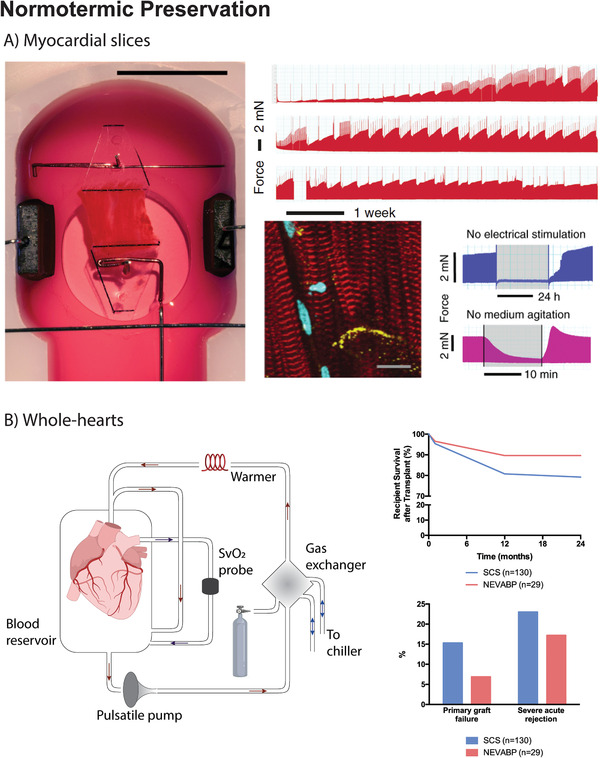
A) In the work by Fischer et al., a new biomimetic culturing technique allowing the long‐term preservation of human myocardial tissue slices under physiological conditions was developed. This system allowed the continuous monitoring of myocardial tissue excitation and contraction, which could be stimulated or inhibited. Long‐term preserved tissue showed dense and well‐aligned myofibrils with preserved cross‐striations (α‐Actinin, red staining) and distinct localization of connexin‐43 (yellow staining). Reproduced under the terms of the CC BY license.^[^ ^34^ ^]^ Copyright 2019, The Authors. B) The publication by Koerner et al., reports a clinical study, where the clinical outcome of patients receiving a heart transplant preserved by normothermic ex vivo allograft blood perfusion (NEVABP) using the Organ Care System by Transmedics, USA, was compared to that of patients receiving a heart transplant preserved by static cold storage. Survival rates at 30 days, 1 year and 2 years after transplant were superior in the NEVABP group, which had a lower incidence of graft failure and severe acute rejection. Figure 4B left panel was created with BioRender.com.

This technology is currently available under the commercial name Organ Care System (TransMedics, USA), where donor hearts can be successfully preserved by ex vivo machine perfusion at normothermia. In their proof‐of‐principle study, porcine hearts were kept in a beating working state for 12 h and compared to hearts preserved for the same time in modified Belzer solution at 4 °C.^[^ ^91^ ^]^ Hearts preserved in normothermia showed increased left ventricular developed pressure, fewer edema and acidosis, and better preservation of coronary endothelial vasomotor function. Later, in a prospective, nonrandomized, single‐institutional clinical study, the clinical outcome of patients receiving a heart transplant preserved by normothermic ex vivo allograft blood perfusion (NEVABP) was compared to that of patients receiving a heart transplant preserved by static cold storage^[^ ^92^ ^]^ (**Figure** **4**B). Cumulative survival rates at 30 days, 1 and 2 years were superior in patients receiving a heart preserved by NEVABP. In addition, graft failure and severe acute rejection were more common in patients receiving a heart preserved by static cold storage.

Altogether, these reports prove that it is possible to safely prolong the preservation period of donor hearts beyond the 4–6 h limit set by static cold storage. This constitutes a significant advance in the field and can potentially contribute to shorten the gap between supply and demand of donor hearts. Yet, the higher complexity and reduced scalability as well as increased costs of this approach represent some of its drawbacks. Of note, future studies should better discriminate the role of temperature and perfusion during the preservation of whole‐hearts by testing hypothermia versus normothermia in the same perfusion regime. Even though NEVABP proved useful, a side‐by‐side comparison with hypothermic perfusion is required to unequivocally determine the best preservation strategy.

Recently, normothermic preservation received additional attention as it was used to study the pathophysiological processes of human cardiac diseases. A new biomimetic, culturing technique consisting of the long‐term preservation of myocardial tissue slices under physiological conditions was developed^[^ ^34^ ^]^ (Figure 4A). The system allows a constant excitation and contraction of myocardial tissue. For that, an exchangeable incubation chamber and a device that generates and monitors electrical stimulations were placed within a regular CO_2_ incubator. Upon constant electrical stimulation, myocardial slices could be preserved for up to 4 months.^[^ ^34^ ^]^ This storage duration could only be achieved when the culture was constantly stirred by a rocker plate, to ensure oxygen supply. One advantage of myocardial tissue slices culturing as opposed to CMs single cultures is sample's multicellularity. Different cell types release functionally important endogenous mediators that promote CM interactions and therefore facilitate tissue contractions upon electrical stimulation. Unfortunately, these contractions could not be generated in the first 2 weeks of culture, which was presumably due to the absence of physiological stimuli, such as catecholamines, and a downregulation of genes involved in the excitation–contraction coupling. Furthermore, all myocardial slices developed bradycardia after preservation as a consequence of the low frequency pacing rate (0.2 Hz), which was imposed to minimize oxygen and nutrient demands.^[^ ^34^ ^]^ Hence, despite representing a significant advance, the use of myocardial slices is still limited by nutrient and oxygen diffusion limit, which was partially addressed by promoting medium agitation. Scaling‐up in slice thickness will most likely depend on tissue perfusion via the preserved vasculature. Similarly, normothermic preservation of cTE constructs, especially of those closely mimicking the native myocardial tissue, will likely depend on efficient perfusion, which can be achieved by increasing cTE construct porosity and/or promoting vascularization (Section 7, a roadmap to cTE construct preservation for more information).

Figure 4 illustrates key publications in the field of normothermic preservation of cardiac samples.

A summary of studies reporting strategies for the preservation of hPSC‐CMs, cardiac tissues and whole‐hearts is provided in **Table** **2**.

**Table 2:** Summary of studies reporting strategies for the preservation of hPSC‐CMs, cardiac tissues and whole‐hearts: cryopreservation, vitrification, normothermic, and hypothermic storage

Cardiac sample	Preservation	Preservation protocol	Major achievements	Ref.
hESC‐CM	Cryopreservation	Sample: Single cells (4–8 × 10^7^ cell per 1.5 mL vial) Cooling rate: 1 °C min^−1^ (37 → −40 °C) + 5 °C min^−1^ (−40 → −80 °C) Freezing medium: CryoStorCS10 Storage time and temperature: Long term; −196 °C (LN_2_)	Cell recovery: 70–77% Quality attributes: electrophysiological phenotype and AP responses; viable cardiac grafts after transplantation into rat MI models	^[^ ^51^ ^]^
	Cryopreservation	Sample: Single cells; Differentiation stage: i) day 12—prebeating vs ii) day 16—post‐beating Treatment: Y‐27632 (10 µmol L^−1^, 1 h, 37 °C) Cooling rate: 1 °C min^−1^ (37 → −70 °C) Freezing medium: 60% RPMI1640 w/B27 +10% DMSO +30% FBS Storage time and temperature: Long term; −196 °C (LN_2_)	Cell recovery: group i) up to 10% recovered beating CM; group ii) no beating CM Quality attributes: beating frequency of thawed cells of group i) was higher than that of nonfrozen CMs. Beating pattern was irregular/arrhythmic; disrupted nuclear membrane and mitochondria more pronounced in group ii)	^[^ ^52^ ^]^
	Cryopreservation	Sample: Single cells; 1–3 × 10^7^ cell mL^−1^ in 1–2 mL vials Freezing rate: Controlled rate program not specified Freezing medium: CryoStorCS10 + 10 × 10^−6^ m Y27632 Storage time and temperature: Long term; −196 °C (LN_2_)	Cell recovery: 84.3 ± 5.2% Quality attributes: cell viability of 85.8 ± 2.2% and 91.6 ± 7.6% cTnT^+^ cells; cells retained action potential characteristics of CM confirmed by electrophysiological analysis	^[^ ^93^ ^]^
hiPSC‐CM	Cryopreservation	Sample: Single cells; 1 × 10^6^ cells in 300 µL Cooling rate: 1 °C min^−1^ (37 → −80 °C) Freezing medium: 90% KOSR + 10% DMSO Storage time and temperature: Long term; −196 °C (LN_2_)	Cell recovery: 50% compared to nonfrozen hiPSC‐CM Quality attributes: cell viability of 95.7 ± 1.4%; cells showed molecular, physiological and mechanical properties of hiPSC‐CMs, with an enrichment in ventricular myocytes when compared to nonfrozen hiPSC‐CM	^[^ ^57^ ^]^
	Cryopreservation	Sample: Single cells; 2–10 × 10^6^ cells mL^−1^ in 1–2 mL vials; Differentiation stage: day 14 – post‐beating Cooling rate: 1 °C min^−1^ (37 → −80 °C). Controlled rate program not specified Freezing medium: i) CryoStorCS10; ii) 90% FBS + 10% DMSO; iii) 90% KOSR + 10% DMSO; iv) STEMdiff cardiomyocyte freezing medium Storage time and temperature: Long term; −196 °C (LN_2_)	Cell recovery: up to 80% in CryoStorCS10, 90% FBS + 10% DMSO and STEMdiff cardiomyocyte freezing medium; up to 85% in 90% KOSR + 10% DMSO Quality attributes: at 7 days after thawing, up to 90% of the preserved cells expressed cTnT and up to 30% expressed MLC2v, regardless the preservation media; at 5 days after thawing, all cells showed a comparable electrophysiological profile.	^[^ ^58^ ^]^
	Cryopreservation	Sample: Single cells; 2 × 10^6^ cells mL^−1^ in 1 mL vial; Differentiation stage: day 23 – post‐beating Cooling rate: 1 °C min^−1^ (37 → −80 °C). Controlled rate program not specified Freezing medium: 90% FBS + 10% DMSO Storage time and temperature: Long term; −196 °C (LN_2_)	Cell recovery: ≈60% Quality attributes: sarcomere length was similar between cryopreserved and fresh cells; RNA sequencing showed an upregulation of cell cycle genes in three cryopreserved hiPSC‐CM lines; three out of five cell lines showed reduced contraction velocity and all lines showed a decrease in contraction deformation after thawing; four out of five recovered hiPSC‐CM lines, showed shortened action potential duration and a propensity to increased beating rate; cryopreserved hiPSC‐CMs have an enhanced propensity for drug‐induced arrhythmias.	^[^ ^61^ ^]^
	Cryopreservation	Sample: Single cells; 1–8 × 10^6^ cell mL^−1^ in 1 mL vial; Differentiation stage: after day 15 of differentiation, from passage 1 to passage 5 of the expansion protocol; Cooling rate: 1 °C min^−1^ (37 → −80 °C) from 4 to 12 h; CoolCell or 2‐propanol‐filled freezing container; Freezing medium: STEMdiff Cardiomyocyte Freezing Medium; Storage time and temperature: Long term; LN_2_ or −150 °C	Cell recovery: cell viability after thawing decreases from passage 1 to passage 5 (70% vs 28%). Quality attributes: hiPSC‐CM maintained their morphology and proliferative capacity when compared to noncryopreserved cells.	^[^ ^94^ ^]^
	Hypothermic storage	Sample: 2D monolayers vs 3D aggregates of hiPSC‐CM Medium: HypoThermosol solution Storage time and temperature: 3, 5, and 7 days; 4 °C	Cell recovery: 2D monolayers‐ 80%, 60% and 50% cell recovery for 3, 5, and 7 days of preservation, respectively; 3D monolayers‐ >70% recovered metabolic activity after 7 days of storage Quality attributes: Cells showed metabolic activity similar to that of the nonpreserved hiPSC‐CMs maintained in culture during the storage period and maintained their typical (ultra)structure, gene and protein expression profile, electrophysiological profiles and drug responsiveness	^[^ ^32^ ^]^
Cardiac Construct	Vitrification	Sample: sheets of human skeletal myoblasts (1 × 10^6^ cell cm^−2^) in polypropylene mesh sheets Protective vehicle: 6.5 m ethylene glycol, 0.7 m sucrose and 10% carboxyl poly‐l‐lysine; 5 min Vitrification time: 5 min Storage time and temperature: up to 3 months; −196 °C (LN_2_)	Quality attributes: Maintenance of cell viability, cell structure including cell–cell junctions such as desmosomes, ECM, and cell membranes and functionality in a rat MI model when compared to fresh cell sheets	^[^ ^62^ ^]^
	Hypothermic storage	Sample: Neonatal rat CMs (1 × 10^6^ cells) mixed with liquid matrix consisting of 10% Matrigel and 0.9 mg mL^−1^ rat tail collagen type I in a custom‐made silicon mold Medium: ChillProtec solution Storage time and temperature: 1–7 days at 4 °C	Quality attributes: ChillProtec solution preserved mitochondrial function and prevented tissue swelling, while promoting CM survival and preservation of their contractility. After 1 day of hypothermia, the number and arrangement of CMs was comparable to untreated controls. However, for prolonged hypothermia of 3 to 6 days a gradual loss of CMs could be observed in the center of the cardiac construct. Maximum contraction force was completely preserved 1 day after hypothermia but gradually decreased to 50–60% after 5 days of hypothermic storage.	^[^ ^86^ ^]^
Myocardial Slices	Normothermic Storage	Sample: human myocardium slices Vehicle solution: Medium 199 supplemented with penicillin/streptomycin, insulin/transferrin/selenite and 2‐mercaptoethanol (50 × 10^−6^ m) Storage time and temperature: up to 4 months at 37 °C, 5% CO_2_, 20% O_2_, 80% humidity, pacing at 0.2 Hz.	Quality attributes: Preservation of tissue function, structure and differentiation in vitro.	^[^ ^34^ ^]^
Whole‐Heart	Vitrification	Sample: rat hearts Vehicle solution and temperature: Perfusion with Custodiol HTK solution followed by perfusion with mCPA (VS55 containing PEG‐coated SPIONs at a concentration of 5 mg_Fe_ mL^−1^). The perfused heart was submerged in mCPA and vitrified in a mechanical freezer at a cooling rate of 15 °C min^−1^, followed by storage in LN_2_ (−196 °C) for 1 week. Nanowarming was performed in an alternating magnetic field (42.5 kA m^−1^ and 278 kHz). SPIONs were flushed by perfusion with Custodiol HTK. Storage time: up to 1 week at −196 °C (LN_2_).	Recovery and Function: Efficient perfusion and clearing of mCPA after vitrification was reported by optical and magnetic particle imaging. Heart functional recovery after vitrification was not evaluated/reported.	^[^ ^72^ ^]^
	Subzero storage (nonfreezing)	Sample: rat hearts Vehicle solution and temperature: five protocols evaluated i) UW at 4 °C; ii) UW at −1.1 to −1.3 °C; iii) UW at −1.1 to −1.3 °C + ice nucleation; iv) UW + AFP I at −1.1 to −1.3 °C + ice nucleation; v) UW + AFP III at −1.1 to −1.3 °C + ice nucleation Storage time: up to 32 h	Recovery and Function: All hearts preserved at −1.3 °C in the presence of AFPs survived. AFPs protected the heart from freezing and improve survival and developed pressure in subzero preservation for 32 h. Coronary flow was higher in group ii) than in AFP groups iv and v	^[^ ^41^ ^]^
	Subzero storage (nonfreezing)	Sample: rat hearts Vehicle solution and temperature: Four protocols evaluated (with and without ice nucleation) i) UW at 4 °C; ii) UW at −1.1 to −1.3 °C; iii) UW + AFP I at −1.1 to −1.3 °C; v) UW + AFP III at −1.1 to −1.3 °C Storage time: up to 24 h	Recovery and Function: Hearts preserved in group ii) with nucleation froze and died. All hearts preserved at −1.3 °C in the presence of AFPs survived. AFPs protect the heart from freezing, improve survival and hemodynamics, and reduce apoptotic cell death	^[^ ^42^ ^]^
	Subzero storage (nonfreezing)	Sample: rat hearts Vehicle solution and temperature: three protocols evaluated (with and without ice nucleation) i) UW at 4 °C; ii) UW at −1.1 to −1.3 °C; iii) UW + AFP III at −1.1 to −1.3 °C Storage time: up to 24 h	Recovery and Function: AFP III prevents freezing of rat hearts in subzero preservation, prolongs ischemic times and improves post‐transplant viability in rat MI models	^[^ ^43^ ^]^
	Hypothermic storage	Sample: rat hearts Vehicle solution: two formulations evaluated i) Celsior solution ii) Celsior solution + HEMO_2_life (1 g L^−1^) Storage time and temperature: 8 h at 4–8 °C	Recovery and Function: Recovery of 45 ± 2% in group i) and 57 ± 1% in group ii). Viability and infarct size measurements were similar between groups. Similar recovery of left ventricular end diastolic pressure and heart rate were attained in both groups. Coronary flow was higher in group ii) compared to group i)	^[^ ^83^ ^]^
	Hypothermic storage	Sample: rat hearts Vehicle solution: Two formulations evaluated i) Celsior solution ii) Celsior solution + linagliptin (0.25–0.75 ng) Storage time and temperature: 9 h at 4 °C	Recovery and Function: supplementation of Celsior solution with linagliptin prevented preservation‐induced cardiac dysfunction and mitigated mitochondrial fragmentation via inhibition of Drp1 phosphorylation.	^[^ ^85^ ^]^
	Hypothermic storage	Sample: mice hearts Vehicle solution: Two formulations evaluated i) Krebs–Hensleit solution ii) Krebs–Hensleit solution + propolis (up to 250 µg mL^−1^) Storage time and temperature: 24 h at 4 °C	Recovery and Function: Propolis supplementation protected hearts during hypothermic preservation by reducing levels of lipids and proteins oxidation; protected tissue integrity and prevented the histopathological damage induced by hypothermic ischemia; reduced ROS generation in isolated cardiac mitochondria.	^[^ ^81^ ^]^
	Normothermic storage	Sample: human hearts Vehicle solution and temperature: hearts were perfused with blood at 37 °C in an Organ Care System (TransMedics,USA) in resting mode; after complete unloading hearts were perfused in an antegrade manner over the aortic root with a median aortic pressure of 40–80 mmHg and an antegrade coronary flow of 1.2–1.5 mL g^−1^ of cardiac weight.	Recovery and Function: Donor hearts preserved with the OCS system were subsequently transplanted. The cumulative survival after transplant was superior in recipients receiving normothermia‐preserved hearts in comparison to standard static cold storage in HTK solution. Primary graft failure and episodes of severe acute rejection were more frequent in the group preserved in static cold storage.	^[^ ^92^ ^]^

AFPs: antifreeze proteins; CMs: cardiomyocytes; cTnT: cardiac troponin T; DMSO: dimethyl sulfoxide; ECM: extracellular matrix; FBS: fetal bovine serum; hESC‐CMs: human embryonic stem cell derived cardiomyocytes; hiPSC‐CMs: human induced pluripotent stem cell derived cardiomyocytes; HTK: histidine‐tryptophan‐ketoglutarate; KOSR: knockout serum replacement; LN2: liquid nitrogen; mCPAs: magnetic cryopreservation agents; MLC2v: myosin regulatory light chain 2—ventricular isoform; SPIONs: superparamagnetic iron oxide nanoparticles; UW: University of Wisconsin solution.

## 5 State of the Art in the Preservation of Cell‐Free Scaffolds

A major difference between heart tissue and cTE constructs is that the latter are generally composed of cardiac cells embedded in a hydrogel that contains ECM‐derived proteins, rather than native ECM. Furthermore, in order to match the native heart mechanics, cTE constructs often present fiber‐based supportive synthetic polymer structures.^[^ ^95^ ^]^ It is therefore crucial to assess the effects of preservation methods on the acellular portion of bioengineered constructs (e.g., hydrogels, ECM proteins, as well as synthetic polymers).

### 5.1 Decellularized Constructs

A study addressing the impact of conventional cryopreservation and vitrification on decellularized bovine pericardium fragments showed that both methods were able to preserve ECM structures, but conventional cryopreservation disturbed its biomechanical properties.^[^ ^96^ ^]^ Both preservation protocols were applied to rectangular scaffolds of 6 cm^2^. For conventional cryopreservation, medium 199 was enriched with 10% DMSO and scaffolds cooled‐down at a slow‐cooling rate of ≈ 1 °C min^−1^. After cooling to −80 °C, scaffolds were plunged into liquid nitrogen and stored in a mechanical freezer set at −150 °C for 1 month. Subsequently, convectional thawing was performed by transferring the samples to a 37 °C water bath. For vitrification, scaffolds were infiltrated in increasing concentrations of precooled VS83 and stored in bags filled with VS83 and covered with 2‐methylbutane to prevent air contact and ice nucleation. Samples were transferred to vapor‐phase nitrogen, which allowed generation of cooling rates of ≈ 20 °C min^−1^. When the scaffold temperature reached −100 °C, bags were relocated to the −150 °C mechanical freezer. Likewise, thawing was accomplished by placing the bags in a 37 °C water bath for 1–2 min. Post‐preservation histologic analysis showed that conventional cryopreservation and vitrification were equally efficient in maintaining ECM structure and components. Collagen bundles and elastin fibers showed regular organization and glycosaminoglycans were present in the expected ratio. However, the slow‐cooled samples possessed altered biomechanical properties, which resulted in atypical stress–strain behavior.^[^ ^96^ ^]^ This alteration was presumably caused by formation of extracellular ice crystals that damaged ECM components, thereby resulting in a decrease in compliance. In conclusion, these results indicate that vitrification might be a preferred method to retain ECM components and functionality.

Another study has reported the long‐term preservation of a decellularized porcine myocardial patch, embedded with synthetic cardiac stromal cell‐secreted factors (synCSCs).^[^ ^97^ ^]^ The transplantation of fresh and cryopreserved (28 days at −80 °C) patches on rat and pig MI hearts equally increased left ventricular EF and cardiac contractility, indicating no excessive alterations in scaffold properties. However, as the protective capacity of the patch is considered to be associated with the pro‐regenerative factors of synCSCs, the effect of cryopreservation remains unclear.^[^ ^97^ ^]^

### 5.2 Hydrogels

Apart from cardiac cells, cTE constructs contain a hydrogel, a material that has a high‐water content.^[^ ^98^ ^]^ In general, a positive correlation can be found between the amount of water present in a cell or structure and the ice‐induced damage during cryopreservation. Adding to the detrimental effects on the embedded cells, freezing can also alter hydrogel flexibility and strength. A recent study has shown that a water‐cryoprotectant binary solvent containing organohydrogel (OHG) could prevent this.^[^ ^98^ ^]^ These OHGs are formed via a displacement approach stimulating the partial replacement of water with a cryoprotectant solution. A europium‐alginate/polyvinyl alcohol (PVA) hydrogel was submerged in a cryoprotective bath containing EG, GC, or d‐sorbitol (SB), and due to osmotic pressure, a large amount of water in the hydrogel was displaced by one of the cryoprotective solutions. The resulting OHGs were resistant to temperatures as low as −45 °C and after 4 h of immersion, they were still bendable, twistable, and foldable at this temperature. These findings represent an important advance and can potentially contribute to the development of improved/cryopreservation‐resistant cTE constructs. Yet, considering the cytotoxic effects of CPAs, further research should determine whether the increased CPA load in OHGs negatively impacts the cellular compartment.

## 6 Promoting Natural Adaptations to Enhance Preservation Protocols

During hypothermic preservation, cells, tissues, and organs naturally adapt to the occurrence of cold‐induced ischemia by decreasing metabolic activity, energy storage and oxygen demand.^[^ ^33^ ^]^ Failure to do so results in extensive cell damage by I/R injury, which, in the heart, translates into reduced post‐preservation myocardial integrity. Hence, boosting these natural adaptations can serve as a strategy to improve post‐storage recovery (**Table** **3**).

**Table 3:** Natural adaptations potentiating hypothermic preservation of cardiac samples

Class	Molecule	Reported biological function	Refs.
Metabolic inhibitors	Hydrogen sulfide (H_2_S)	Inhibits inflammation and halts apoptosis (inactivation of NF‐κB)	^[^ ^30^ ^]^
		Activates antioxidants (SOD, CAT, and GSH)	^[^ ^100^ ^]^
Mitochondria respiration inhibitors	Rotenone	Irreversible inhibitor of complex I; lowers ROS levels	^[^ ^101^ ^]^
	Amobarbital	Reversible inhibitor of complex I; controls mitochondrial damage	^[^ ^102^ ^]^
	Metformin	Inhibitor of complex I; increase mitochondrial calcium retention	^[^ ^103^ ^]^
Stress tolerance enhancers	Y‐27632	Inhibitor of the pro‐apoptotic protein Rho‐associated kinase (ROCK)	^[^ ^54^ ^]^
	Exendin‐4	Inhibits apoptosis and oxidative stress	^[^ ^104^ ^]^
Stress survival pathway stimulators	Erythropoietin (EPO)	Increases phosphorylation of STAT3; activates SAFE pathway	^[^ ^105^ ^]^
	Glyceryl trinitrate	Activates SAFE pathway	^[^ ^105^ ^]^
	Zoniporide	Activates SAFE pathway	^[^ ^105^ ^]^
	Human recombinant neuregulin‐1 peptide (rhNRG‐1)	Activates RISK and SAFE pathways	^[^ ^106^ ^]^
Sugars	Fructose‐1,6‐bisphosphate (FBP)	Glycolytic substrate	^[^ ^107^ ^]^
		Calcium chelator	^[^ ^107^ ^]^
		Activates phosphofructokinase‐1	^[^ ^108^ ^]^
		Upregulates pentose phosphate pathway	^[^ ^109^ ^]^
Hormones	Dopamine	Lowers lactate dehydrogenase (LDH) release and increases ATP regeneration	^[^ ^110^ ^]^
	*N*‐octanoyl dopamine (NOD)	Lowers lactate dehydrogenase (LDH) release and increases ATP regeneration	^[^ ^110^ ^]^

In line with this, it is anticipated that samples preserved under hypothermic conditions can benefit from the presence of a wide range of metabolic inhibitors. For example, hydrogen sulfide (H_2_S) can decrease damage caused by ischemia and reperfusion.^[^ ^30^ ^]^ H_2_S inhibits inflammation, halts apoptosis via the inactivation of NF‐κB, and activates antioxidants (superoxide dismutase (SOD), catalase (CAT), and reduced glutathione (GSH)).^[^ ^99^, ^100^ ^]^ One study showed that a slow‐releasing H_2_S system (DATS‐MSN) alleviated myocardial injuries if incorporated during the hypothermic preservation of rat hearts.^[^ ^100^ ^]^ The system is based on mesoporous silica nanoparticles carrying diallyl trisulfide. After activation by GSH, the nanoparticles release H_2_S into the preservation medium. During 6 h of rat heart preservation in UW solution at 4 °C, the DATS‐MSN system could slowly enrich the medium with H_2_S. Hearts preserved in the presence of DATS‐MSN exhibited improved function, including elevated left ventricle developed pressure and d*p*/d*t*_max_ as well as lower arrhythmia scores. Moreover, mitochondrial structure and function were better preserved in the presence of DATS‐MSN, as shown by transmission electron microscopy, lower leakage of cytochrome *c* from the mitochondria to the cytoplasm, and increased myocardial ATP content. Eight weeks after heterotopic transplantation, hearts that had been preserved in the presence of DATS‐MSN showed improved survival and function (i.e., EF and fractional shortening) and reduced fibrosis in comparison to the control and other H_2_S releasing alternatives. Mechanistically, increased H_2_S levels by DATS‐MSN produced an anti‐inflammatory effect by inhibition of the TLR4/NLRP3 signaling pathway. Also, by protecting mitochondrial structure and function, H_2_S prevented cardiomyocyte apoptosis.^[^ ^100^ ^]^

A variety of metabolic inhibitors decrease ischemia‐induced mitochondrial damage by blocking the electron transport chain. For example, administration of rotenone, an inhibitor of complex I, lowered ROS levels after 45 min of ischemia.^[^ ^101^ ^]^ While interesting, these results have poor translatability given the irreversible nature of the inhibition. Hence, cardio‐protection granted by reversible inhibitors is considered to be more clinically relevant. Amobarbital is able to reversibly bind the same site as rotenone, resulting in mitochondrial damage control after 25 min of ischemia in rat hearts.^[^ ^102^ ^]^ While both studies describe protection against ischemia, mitochondrial damage can also occur during reperfusion. In line with this, cardio‐protection via metformin‐mediated complex I inhibition was tested in an ischemia/reperfusion murine model.^[^ ^103^ ^]^ Acute treatment with high dose of metformin after ischemia limited infarct size. This event correlated with increased mitochondrial calcium retention, indicating a reduced opening of the permeability transition pore.^[^ ^103^ ^]^

An alternative strategy to prolong preservation time of cardiac tissues may be the use of stress tolerance enhancers, of which antioxidants and apoptosis inhibitors are key components. For example, ROCK inhibitor Y‐27632 is routinely used during cryopreservation and hypothermic preservation.^[^ ^32^, ^53^, ^54^ ^]^ Using Exendin‐4 may also hold potential for cardio‐protection during preservation as it was shown to inhibit apoptosis and oxidative stress in the infarcted myocardium and contributed to better preservation of cardiac function (i.e., higher left ventricle systolic pressure and d*p*/d*t*_max_ and lower left ventricle end‐diastolic pressure) in a murine model of I/R injury.^[^ ^104^ ^]^

Several other studies have tested the impact of stress survival pathway stimulation in the response to I/R injury during hypothermic preservation. For example, the cardioprotective effect of erythropoietin (EPO) supplementation during hypothermic storage media was demonstrated.^[^ ^105^ ^]^ EPO and other stress‐response activating additives, were added to Celsior solution while hearts were stored on ice for 6 or 10 h. After 6 h of storage, hearts preserved in the presence of EPO showed improved recovery of aortic flow, coronary flow, cardiac output and heart rate in a dose‐dependent manner. EPO administration was associated with increased phosphorylation of STAT3, indicating a possible role of EPO as an activator of the survivor‐activating factor enhancement (SAFE) cytoprotective pathway. Interestingly, a synergistic combination of EPO, glyceryl trinitrate, and zoniporide, all activators of the SAFE pathway, could expand cardiac protection up to 10 h of hypothermic storage.^[^ ^105^ ^]^ Earlier, the same experimental‐setup was used to elucidate the protective effect of recombinant human neuregulin (rhNRG)‐1 during prolonged hypothermic cardiac preservation.^[^ ^106^ ^]^ When added to Celsior, rhNRG‐1 improved the functional recovery (i.e., cardiac output, heart rate, and aortic and coronary flow recovery) of hearts preserved for 6 h at 4 °C compared to control, through the activation of SAFE and reperfusion injury salvage kinase (RISK) pathways. Interestingly, the combined action of rhNRG‐1, glyceryl trinitrate and cariporide allowed further protection against I/R injury caused by hypothermia of up to 10 h.^[^ ^106^ ^]^

Moreover, several additives have been reported to aid cardiac preservation despite the lack of information on the exact working mechanism. An example was fructose‐1,6‐bisphosphate (FBP), a compound known to enhance the preservation of isolated CMs^[^ ^107^ ^]^ and the functional recovery of rat hearts post‐hypothermic storage.^[^ ^108^, ^109^ ^]^ Several possible mechanisms of action were proposed. Firstly, FBP could be used as glycolytic substrate bypassing the two prior ATP‐consuming phosphorylation steps. Secondly, FBP would have the capacity of chelating Ca^2+^, resulting in a lower extracellular, and subsequently intracellular, Ca^2+^ concentration. Lower Ca^2+^ concentrations demand less ATP consumption for Ca^2+^ transport and therefore delay energy depletion. Thirdly, it was suggested that FBP allosterically activates phosphofructokinase‐1, resulting in an increase in glycolysis and therefore ATP production. Lastly, FBP supplementation would lead to an upregulation of the pentose phosphate pathway. In vitro supplementation with FBP was accompanied by a reduced intracellular Ca^2+^ concentration in CMs maintained at 3 °C.^[^ ^107^ ^]^ Such results support the hypothesis that Ca^2+^ chelation by FBP is an additional cardioprotective mechanism. Given the participation of Ca^2+^ in many signaling pathways, further research is needed to identify the molecular players governing the protective role of FBP in cardiac cells and tissue preservation.

Inspired by the observation that donor treatment with low‐dose dopamine improves clinical outcome after heart transplant, the effect of dopamine and *N*‐octanoyl dopamine (NOD), a dopamine derivate, was tested during the hypothermic preservation of CMs.^[^ ^110^ ^]^ First, neonatal rat CMs were treated with either dopamine or NOD before exposure to static cold storage for 8–12 h. Afterward, rewarming was achieved by convection in a 37 °C water bath. Compared to untreated cells, treated cells released less LDH during cold storage, indicating reduced CM death. Additionally, the cellular ATP content was higher within the treated groups. Both LDH decrease and ATP increase were most significant after NOD administration compared to dopamine addition. After rewarming, treated CMs were also able to retrieve regular ATP levels, while untreated cells did not regenerate their ATP content. In addition, 94% of all NOD‐treated CMs regained spontaneous contractions after rewarming, compared to 89% of the dopamine‐treated cells. In an attempt to translate these results to a more complex system, rat hearts perfused with NOD before explantation were shown to release less LDH after hypothermic preservation, despite no beneficial effects were reported at the metabolic or functional level.^[^ ^110^ ^]^

## 7 A Roadmap to cTE Construct Preservation

**Figure 5:**
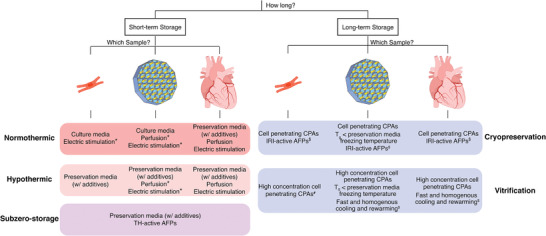
Synopsis of strategies for preservation of cardiac cells, cTE constructs and hearts. *: Not required/optional. $: Considered useful but not experimentally tested/reported. #: Technically possible but less preferred compared to conventional cryopreservation. AFPs: antifreeze proteins. TH: thermal hysteresis. CPAs: cryoprotective agents. IRI: ice recrystallization inhibition. *T*_g_: glass‐transition temperature. Created with BioRender.com.

To date, no research has focused on or provided an optimized protocol for the storage of cTE constructs. Yet, lessons can be taken from currently available methodologies. A first critical/defining step relates to the temporal need of preservation. Long‐term preservation techniques, such as cryopreservation, can preserve a specimen without deterioration for months or years, allowing off‐the‐shelf clinical availability. On the other hand, when shorter preservation periods are required (i.e., hours/days), specimens can be preserved in a range of normothermic to subzero temperatures, avoiding the ice (re)crystallization and CPA toxicity limitations. Short term preservation may suffice when specimens have to be transported between hospitals and/or research facilities (**Figure** **5**).

A second critical aspect in the preservation of cTE constructs relates to its size/thickness. Currently developed cTE constructs are composed of cardiac cells, an ECM‐containing hydrogel and often a fiber‐based synthetic polymer structure, which altogether provide a functional and mechanically stable construct that can, depending on its size, assist in the contraction of the damaged heart upon transplantation. However, increased size/thickness proportionally hinders the diffusion of protective molecules, such as CPAs, inside the construct. This is a point of contrast with native‐tissue preservation as the latter possesses a well‐organized vasculature that can be used as the delivery route of protective CPAs. In line with this, different strategies have been considered to increase the porosity of cTE constructs, but this will likely affect its mechanical and functional (e.g., electrical) properties,^[^ ^111^ ^]^ as cell–cell contacts and tissue morphology are altered. As such, increased cTE construct complexity, e.g., constructs composed of multiple cell types, densely organized into a native tissue‐like architecture, will benefit from a tailored preservation approach. Currently, three classes of cTE constructs can be distinguished: thin (<300 µm) composed of a cell sheet or monolayer^[^ ^112^ ^]^; thick (>300 µm) achieved by stacking multiple cell sheets or use of scaffolds; and advanced with higher complexity (>300 µm) where the diffusion of molecules is increased by vascularization^[^ ^113^, ^114^, ^115^ ^]^ or forced flow of medium.^[^ ^116^ ^]^ Those exhibiting a close‐to‐native thickness (i.e., 5.3 to 9 mm)^[^ ^117^ ^]^ may hold the greatest clinical potential, as these are expected to provide the strongest contractile support, although the force produced by a construct does not merely depend on its size, but also on cell density, maturity, and organization. Furthermore, a novel class of advanced large cTE constructs is currently in development, so‐called biological ventricular assist devices, capable of wrapping part of the heart to support ventricular contractility (Horizon2020 BRAV∃).^[^ ^118^ ^]^

Considering these different construct types, the roadmap to cTE construct preservation will not depict a one‐size‐fits‐all approach but will provide several directions. cTE construct preservation techniques will have to be optimized to meet the specific requirements of each cTE construct. Those closely resembling the native tissue in size and/or complexity will require strategies that simultaneously grant whole‐organ preservation. In contrast, simpler and thinner cTE constructs may be efficiently preserved with techniques proving useful for tissue slices or even for monolayers/3D aggregates of cardiac cells. For thick constructs that are obtained by stacking several cell sheets or thin constructs, the individual layers could be preserved separately using a less complex strategy and assembled into thicker constructs after thawing.

Conventional cryopreservation was optimized to prevent the formation of intracellular ice crystals but extracellular ice crystallization may occur. As cell–cell and cell–matrix interactions are essential for the normal functioning of tissues, these can be significantly compromised by extracellular ice development during conventional cryopreservation and even hamper post‐thaw recovery.^[^ ^80^ ^]^ Similarly, large, complex and multicellular cTE constructs will likely be affected by extracellular ice formation. IRI‐active AFPs are capable of inhibiting ice recrystallization and may allow the preservation of complex samples without sharp and big ice crystals being formed.^[^ ^66^ ^]^ Although very promising, no studies have addressed the use of AFPs for cryopreservation of cardiac native or engineered tissues.^[^ ^119^ ^]^ In addition, other macromolecular polymers (e.g., PVA) and polyampholytes) are currently under investigation for the use in cryopreservation of biological samples.^[^ ^120^, ^121^ ^]^ Supplementation of cryopreservation media either with PVA^[^ ^122^ ^]^ or with the polyampholyte poly(ε‐lysine)^[^ ^123^ ^]^ improved cell recovery after cryopreservation. While PVA is an AFP‐inspired synthetic polymer with IRI activity,^[^ ^122^ ^]^ cryoprotection by polyampholytes does not seem to rely on IRI alone and is yet to be determined.^[^ ^121^ ^]^ Further research should explore the potential of these natural and synthetic antifreezes for the cryopreservation and functional recovery of cardiac cells and tissues.

Given its capacity of preventing intracellular and extracellular ice formation, vitrification became preferred for cryopreservation of complex samples.^[^ ^19^ ^]^ Clinically relevant cTE constructs exceed the maximum size for convective 37 °C water bath thawing following vitrification. Matching the shape of the sample container to the sample's outline during rewarming or resorting to nanowarming, using msIONPs, sIONPs or SPIONs may prove useful to provide fast and homogenous warming of cTE constructs after vitrification.^[^ ^72^, ^73^, ^75^, ^76^ ^]^ Furthermore, vitrification of cTE constructs relies on a vitrification solution and protective additives. Data from previously conducted studies suggest that DMSO is a preferable CPA for cardiac cryopreservation.^[^ ^70^, ^71^ ^]^ To ensure safe construct preservation, additional protective additives may be required, which raises the question of the delivery route. While in a whole‐organ setting, perfusion with CPAs and additives seems to be the most reliable strategy of delivery, in cTE constructs, additives may be included in the formulation of the hydrogel, provided that it does not affect its functionality. Independently of the preserved specimen, the duration of the incubation with CPAs and other additives should consider the trade‐off existing between homogenous distribution, granting enough cryoprotection, and toxicity induced by CPAs.^[^ ^50^ ^]^ Also, cell retention during cryopreservation was shown to be negatively affected when polymer contraction was higher than cell shrinkage during the freezing phase of fiber scaffolds. Polymer temperature‐induced contraction is related to its crystallinity;^[^ ^65^ ^]^ thus, it is crucial to select polymers with a *T*_g_ lower than the preservation temperature.

Short‐term preservation of cardiac constructs also requires a preservation solution and protective additives. Celsior and UW are the most frequently used protective solutions during hypothermic preservation of myocardial samples.^[^ ^83^ ^]^ Despite UW solution has been reported to provide superior results for the preservation of cardiac tissue^[^ ^31^ ^]^ it is not approved by the Food and Drug Administration (FDA) for cardiac preservation, hence, its use in donor hearts is considered off label.^[^ ^124^ ^]^ Furthermore, based on previous results several additives are considered to hold potential for enhancing hypothermic preservation of cTE constructs, including: Y‐27632 to enhance stress tolerance;^[^ ^32^ ^]^ EPO^[^ ^105^ ^]^ and rhNRG‐1^[^ ^106^ ^]^ to maximize survival; HEMO_2_life to prevent hypoxia establishment;^[^ ^83^ ^]^ linagliptin^[^ ^85^ ^]^ or propolis^[^ ^81^ ^]^ to prevent ROS formation; H_2_S to reduce metabolic activity and I/R injury even further,^[^ ^100^ ^]^ and TH‐active AFPs that allow a further reduction of preservation temperature and, consequently, metabolic activity, expanding preservation duration without I/R injury.^[^ ^40^, ^66^ ^]^ Of note, systematic studies are still required to closely inspect the contribution of each of these additives and determine their usefulness for hypothermic preservation of cTE constructs.

Studies describing the hypothermic storage of cardiac tissues show a limited preservation of 4 to 24 h, which is insufficient for international transport. Yet, iPSC‐CMs 3D‐aggregates were efficiently preserved and recovered after a 7‐day lasting hypothermia,^[^ ^32^ ^]^ which suggests that cTE preservation might be feasible.

Apart from cryopreservation and hypothermic preservation, normothermic preservation with constant perfusion and electrical stimulation can be a valid alternative. This method expanded the ex vivo preservation of whole‐hearts up to 12 h and improved the clinical outcome of patients upon transplantation.^[^ ^91^, ^92^ ^]^ Moreover, myocardial slices could be preserved for 4 months despite some constrains like the compulsory establishment of slow pace stimulations and development of bradycardia as well as a two‐week adaptation period.^[^ ^34^ ^]^ Hence, despite technically and logistically complex, normothermic preservation proved to be an excellent method to preserve complex cardiac samples that fail to recover from prolonged hypothermia. As such, preservation of clinical‐grade cTE constructs resembling the endogenous myocardium may require a stable close‐to‐physiological environment at normothermia. Additional studies are necessary to determine whether this strategy can successfully preserve cTE constructs without significant deterioration for a minimum of two days, allowing international transport and clinical availability.

## 8 Conclusion

Taken together, experiments on the preservation of CMs, heart tissue, tissue constructs, whole‐heart, and even other organs, like the kidneys, provide highly useful insights into the development of specific techniques for storing and preserving cTE constructs. However, lack of empirical data on cTE construct preservation raises an extra layer of questions. How does successful preservation of cTE constructs affect cell maturation and cardiac (functional) integration after implantation? A thorough analysis on existing preservation methods provides a theoretical foundation for cTE construct preservation. Based on recent developments, it is predicted that vitrification of cardiac constructs would be the most promising approach for long‐term storage. On the other hand, hypothermic or even normothermic storage, could facilitate short‐term storage and transport, thereby greatly increasing construct accessibility. Given the noncurative nature of current therapies, implementation of bioengineered tissues to regenerate/repair the heart is expected to have great socio‐economic impact by preventing the development of HF and improving patient's quality of life.

## Conflict of Interest

L.W.v.L. reports consultancy fees to UMCU from Abbott, Medtronic, Vifor, Novartis, and research materials from Roche and Sopachem (all outside of the current work).
